# Supplementary material for: Identification of genomic enhancers through spatial integration of single‐cell transcriptomics and epigenomics
Source: Mol Syst Biol. 2020 May 19;16(5):e9438. doi: 10.15252/msb.20209438 (PMC7237818; doi:10.15252/msb.20209438)
Supplement: Supplementary file 1 — Appendix [file MSB-16-e9438-s001.pdf]

Table of contents

Figure S1. Quality control and marker genes from the scRNA-seq data set (3,531 cells).
Figure S2. Identification of photoreceptor subclasses and cone cells in the ommatidial cells.
Figure S3. Comparison with a public Drop-seq data set from the eye disc with 11,500 single cell profiles.
Figure S4. Selection of high-quality cells using CellRanger and CellRanger ATAC.
Figure S5. Validation of the gene regulatory networks in the morphogenetic furrow.
Figure S6. Quality control of scATAC-seq data.
Figure S7. Selection of regulatory regions for the analysis of scATAC-seq data.
Figure S8. Cell clustering is driven by cell type and not by batch effects.
Figure S9. Motif enrichment on the regions within regulatory topics reveal key master regulators of the cell populations in the eye-antennal disc.
Figure S10. Annotation of topics and cell clusters from scATAC-seq profiles.
Figure S11. Validation of cell type annotations using previously published FAC sorted single-cell ATAC-seq data.
Figure S12. Derivation of gene activity scores using scATAC-seq data and comparsion with gene expression.
Figure S13. The size of the virtual template has no impact in our results.
Figure S14. Ordering cell types in the eye and antenna by pseudotime.
Figure S15. Wide Janelia regions contain multiple enhancers.
Figure S16. Janelia regions lowly correlated with activity correspond to primed enhancers.
Figure S17. Uncoupling enhancer accessibility and activity in validated atonal enhancers from Aerts *et al*.
Figure S18. Enhancer-to-target links support basic principles of gene regulation.
Figure S19. Gene expression is regulated by complex combinations of enhancers.
Fig S20. Enhancer-to-target genes recapitulate key aspects of gene regulation.
Figure S21. Genes are regulated by many enhancers, some of which show redundant activity (and accessibility).
Figure S22. Hth is potentially repressed by an enhancer bound by so and also accessible in FAC-sorted Optix-GFP$^+$ cells.
Figure S23. Link-based regulons identify additional master regulators during the eye-antennal disc development.
Figure S24. Cell-type specific analysis of chromatin accessibility QTLs reveals additional motifs involved chromatin opening compared to bulk analysis.
Figure S25. Overexpression of 13 TFs using the GMR-GAL4/UAS-TF system reveals 8 master regulators whose overexpression causes defects during the development of the eye disc
Figure S26. Pros-L overexpression result on the opening of GGG enriched regions.
Figure S27. Repressed enhancers show a reduced ATAC-seq signal.

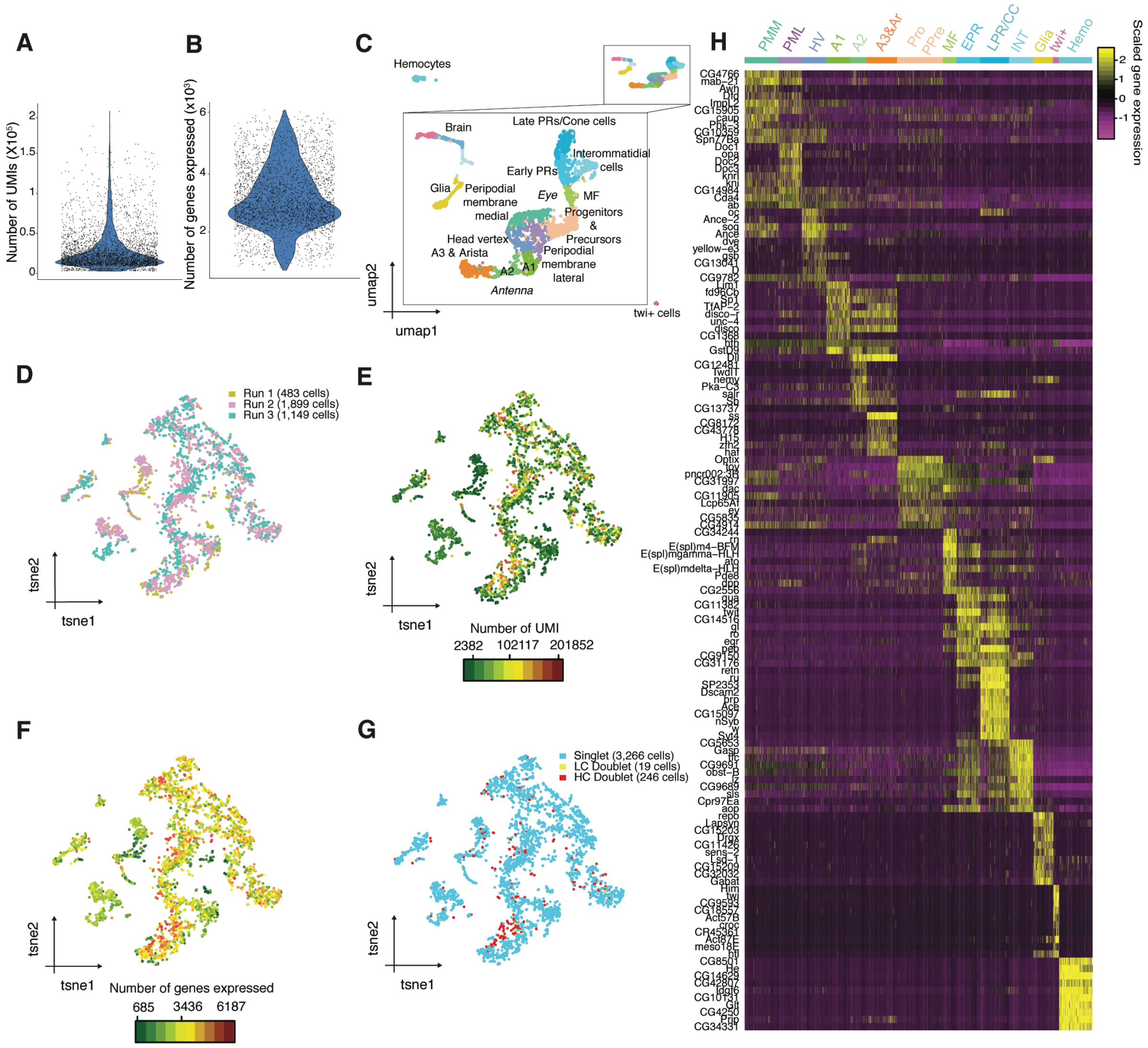

Figure S1. Quality control and marker genes from the scRNA-seq data set (3,531 cells). A. Number of UMIs (Unique Molecular Identifiers) per cell. **B.** Number of genes expressed per cell. **C.** Seurat UMAP (3,531 cells) colored by cell type, with a zooming in for the cell types distinct of hemocytes and twi$^+$ cells. **D.** Seurat tSNE colored by experimental run. **E.** Seurat tSNE (3,531 cells) colored by the number of UMIs per cell. **F.** Seurat tSNE (3,531 cells) colored by the number of UMIs per cell. **G.** Seurat tSNE colored by singlets, low confidence (LC) doublets and high confidence (HC) doublets as defined by DoubletFinder (McGinnis et al., 2019). **H.** Heatmap showing the scaled expression of the top 10 Seurat markers based on differential expression adjusted p-value per group per cell.

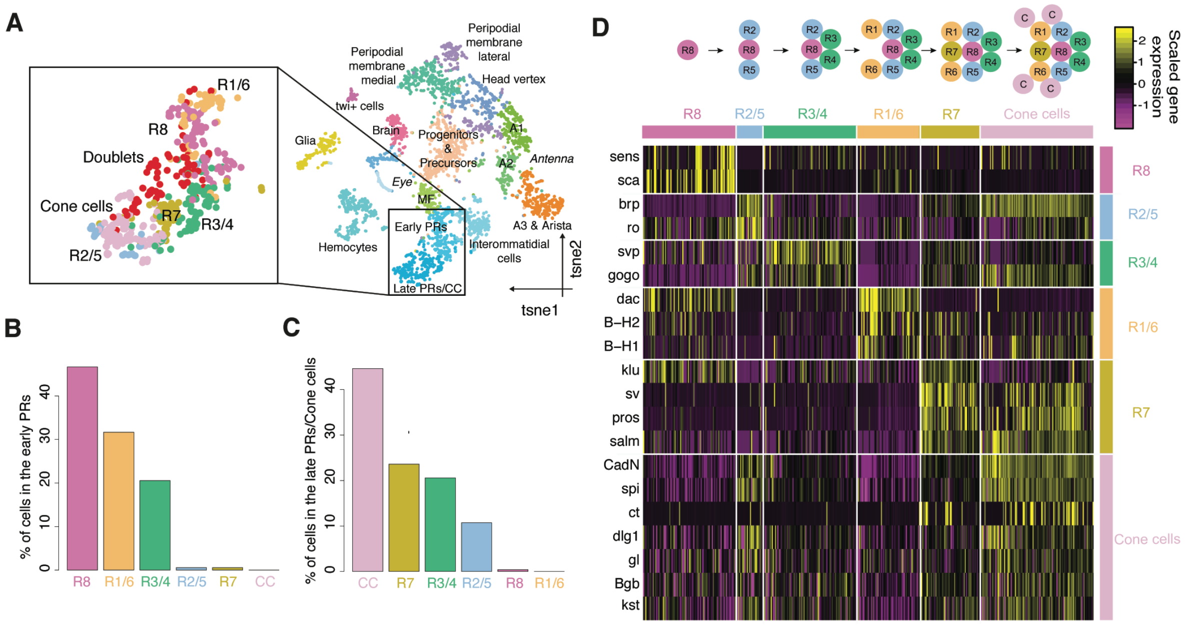

Figure S2. Identification of photoreceptor subclasses and cone cells in the ommatidial cells. A. Seurat tSNE annotated by cell type, showing the interommatidial cell clusters annotated by subpopulations augmented. **B.** Percentage of each of the subpopulations in the cluster labeled as early PRs (photoreceptors). **C.** Percentage of each of the subpopulations in the cluster labeled as late PRs/Cone cells. **D.** Heatmap showing known marker genes for each ommatidial subclass. The ommatidial formation process is shown over the heatmap.

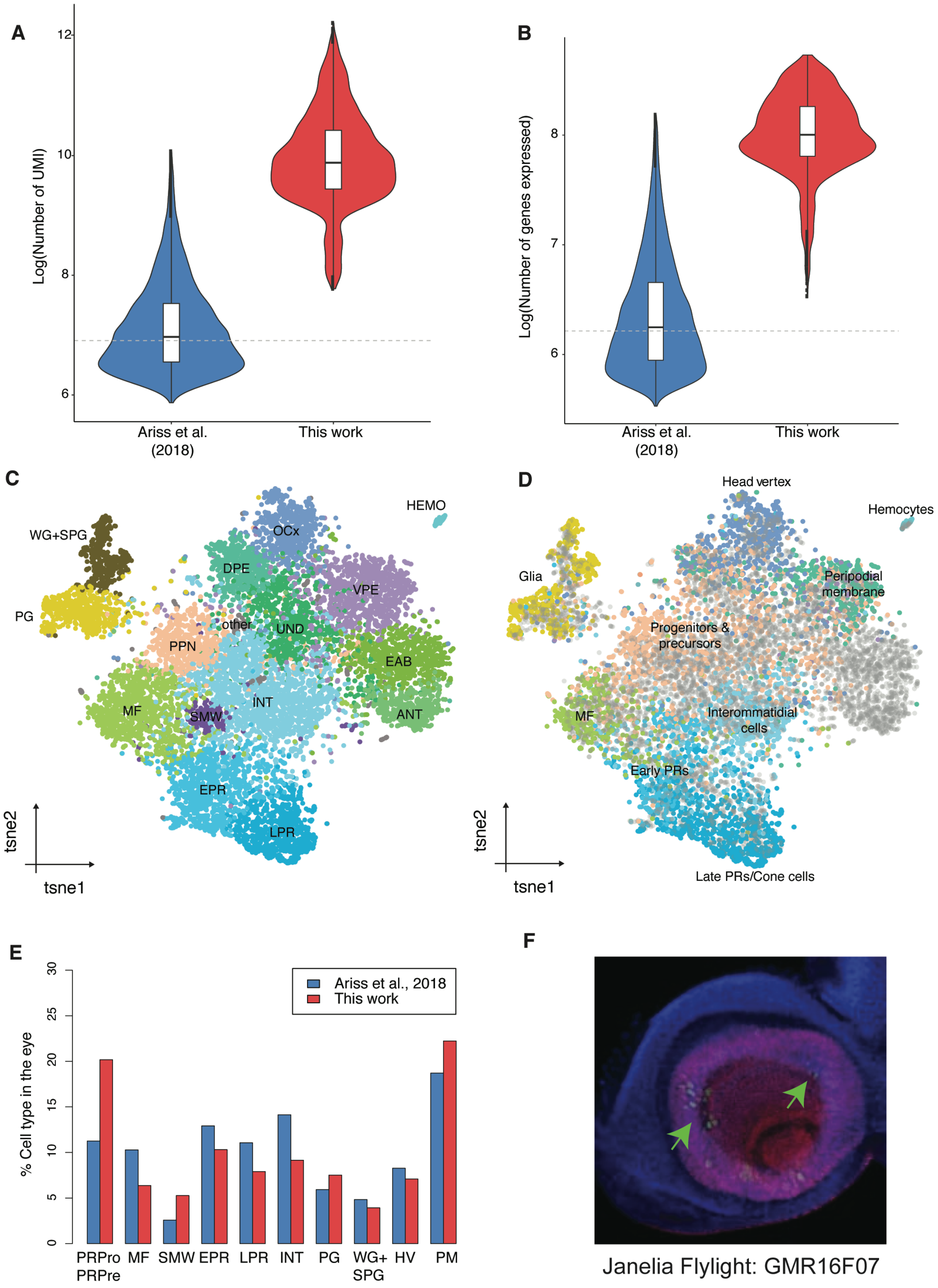

Figure S3. Comparison with a public Drop-seq data set from the eye disc with 11,500 single cell profiles. A. Normalized UMI counts per cell in Ariss *et al.* (2018) (Ariss et al., 2018) and this work. **B.** Normalized number of genes expressed per cell in Ariss et al. (2018) and this work. **C.** Seurat tSNE

annotated by cell type, using the tSNE coordinates provided by Ariss *et al*. (2018). Ar: Arista. CC: Cone cells. EPR: Early photoreceptors. Hemo: Hemocytes. HV: Head Vertex. INT: Interommatidial cells. LPR/CC: Late photoreceptors and cone cells. MF: Morphogenetic furrow. PG: Perineurial glia. PML: Peripodial membrane lateral. PMM: Peripodial membrane medial. PR: Photoreceptors. PRPre: Photoreceptor precursors. PRPro: Photoreceptor progenitors. SMW: Second mitotic wave. SPG: Subperineurial glia. WG: Wrapping Glia. **D.** Seurat tSNE from Ariss et al., colored by label transfer from our data set. Cells in grey were removed from the analysis due to low depth. **E.** Proportion of cell types in the eye disc based on the clusters defined by Ariss et al. (2018) in the original data set and in this work's eye disc data after label transferring with Seurat (Stuart et al., 2019). **F.** Image retrieved from Janelia Flylight (Jory et al., 2012) showing the activity of the enhancer linked to twi, marker of adepithelial cells, in the antennal disc (marked with arrow heads).

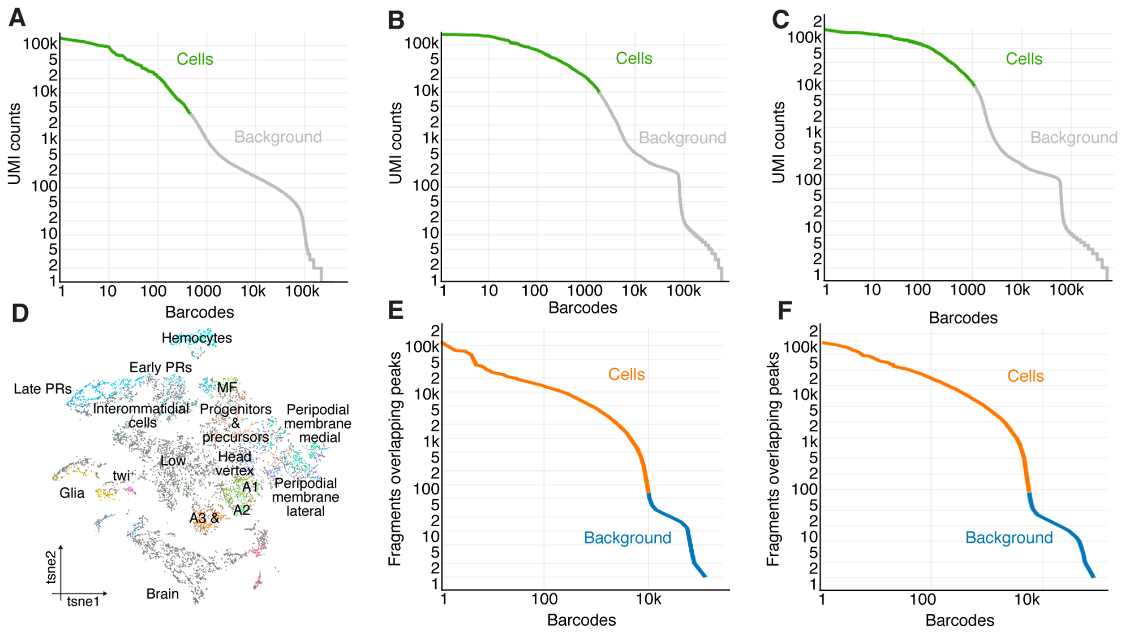

Figure S4. Selection of high-quality cells using CellRanger and CellRanger ATAC. **A.** CellRanger plot showing the number of UMI counts per barcode in the first scRNA-seq run. In total, 483 high-quality cells were selected**. B.** CellRanger plot showing the number of UMI counts per barcode in the second scRNA-seq run. In total, 1,899 high-quality cells were selected. **C.** CellRanger plot showing the number of UMI counts per barcode in the third scRNA-seq run. In total, 1,149 high-quality cells were selected. **D.** Seurat tSNE including 9,234 cells with at least 1,000 UMI counts and 500 genes expressed. Cells not included in our high-quality data set of 3,531 cells are shown in grey. Our data set of 3,5K cells is only a subsample of the entire tissue (estimated to have 15,000-20,000 cells), yet we find a similar cellular composition as in the previous Drop-seq study on the eye disc; and are able to recover rare populations in the antennal disc, such as the Johnston Organ Precursors and the *twi*$^{+}$ adepithelial cells (which comprise less than 1% of the cell population, respectively). **E.** CellRanger ATAC plot showing the number of fragments within aggregate peaks per barcode in the first scATAC-seq run. In total, 9,833 high-quality cells were selected. **F.** CellRanger ATAC plot showing the number of fragments within aggregate peaks per barcode in the second scATAC-seq run. In total, 5,554 high-quality cells were selected.

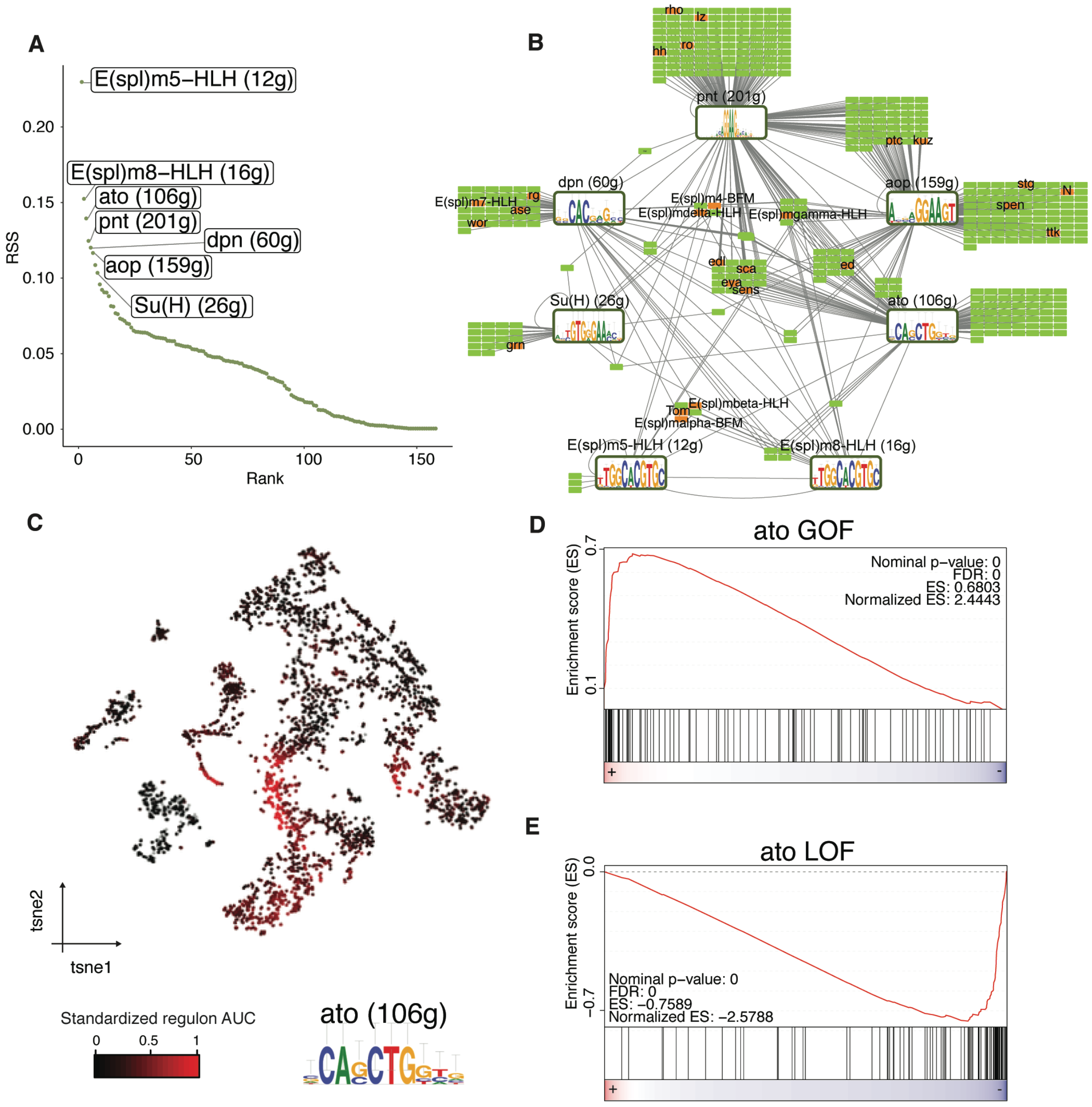

Figure S5. Validation of the gene regulatory networks in the morphogenetic furrow. A. Regulon Specifity Score (RSS) for the morphogenetic furrow. The top 7 regulons are highlighted. **B.** Gene Regulatory Network of the morphogenetic furrow based on the top 7 regulons. Target genes that have associations in GeneMANIA with their TF are shown in orange with their names. **C.** Seurat tSNE (3,531 cells) coloured by the enrichment of the atonal regulon. The top enriched atonal motif on the regulon is shown. **D.** GSEA plot showing the enrichment of the predicted atonal targets on an atonal gain-of-function (GOF) mutant. The gene ranking is based on the logFC from the differential gene expression analysis of the atonal gain-of-function mutant versus wild type (Aerts et al., 2010). **e.** GSEA plot showing the enrichment of the predicted atonal targets in an atonal loss-of-function (LOF) mutant. The gene ranking is based on the logFC from the differential gene expression analysis of the atonal loss-of-function mutant versus wild type (Aerts et al., 2010).

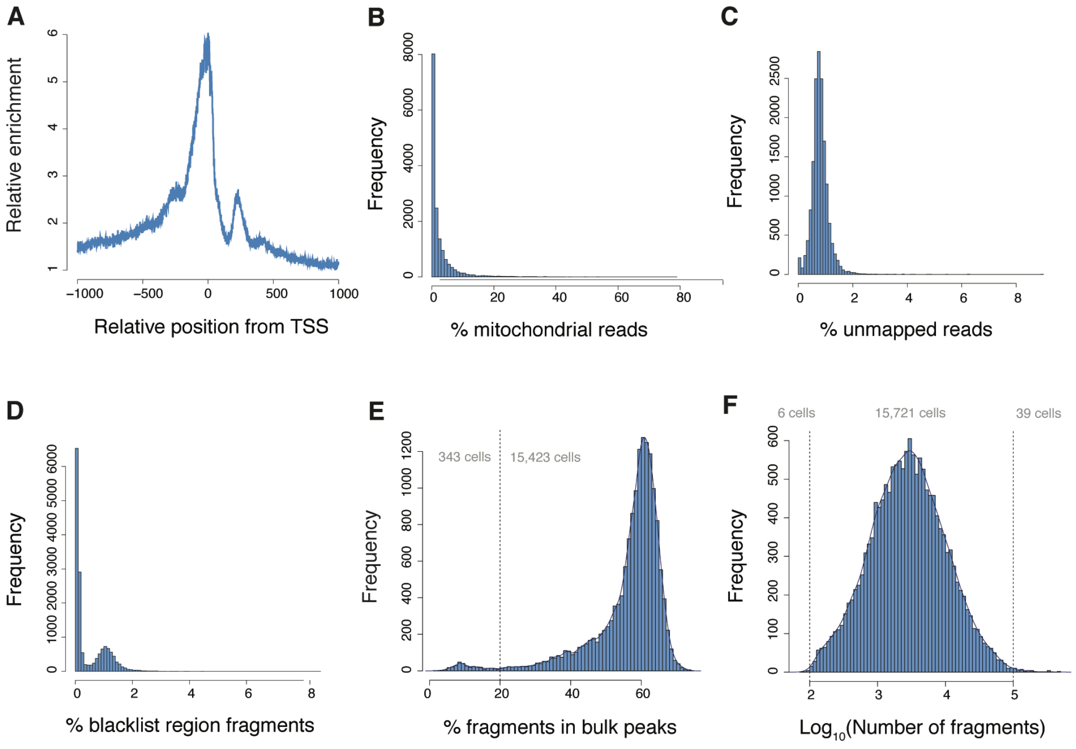

Figure S6. Quality control of scATAC-seq data. A. Relative enrichment of the accessibility signal versus position from the TSS. TSS has higher relative accessibility compared to surrounding areas. **B.** Percentage of mitochondrial reads. **C.** Percentage of unmapped reads. **D.** Percentage of fragments in blacklisted regions. **E.** Percentage of fragments in bulk peaks. Cells with less than 20% of the fragments in bulk peaks, were filtered out. **F.** Normalized (log_{10}) number of fragments. Cells with less than 100 fragments or more than 100,000 fragments were filtered out.

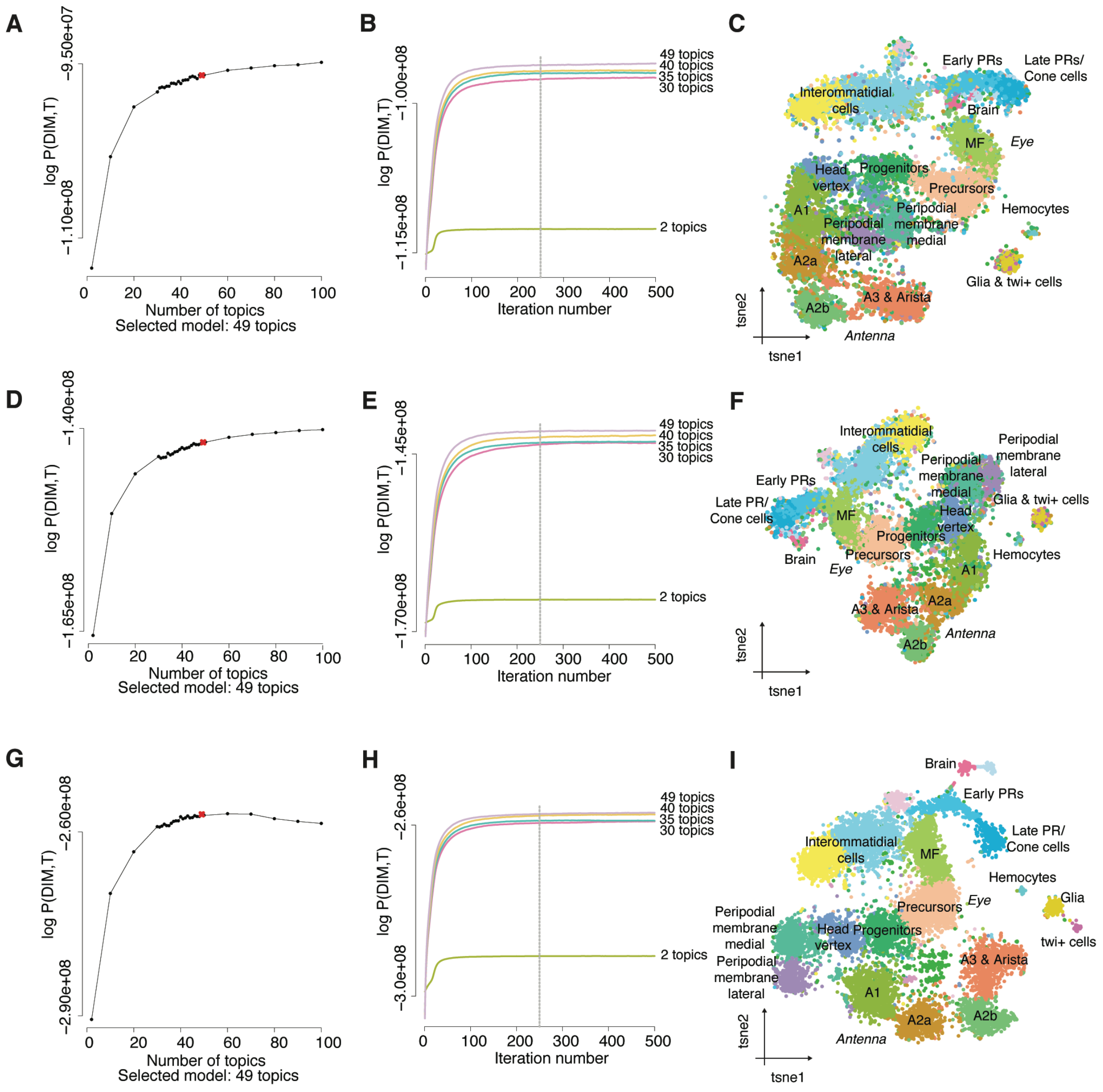

Figure S7. Selection of regulatory regions for the analysis of scATAC-seq data. A-C. cisTopic modelling using bulk narrow peaks as called by MACS as regulatory regions (13,567 regions). The log-likelihood per model, the log-likelihood per iteration for selected models, and the cisTopic cell tSNE (with 15, 387 cells) of the selected model (with 49 topics) are shown. In cisTopic, the log-likelihood in the last iteration is used as criteria for model selection (the model with the highest log-likelihood is taken), and the log-likelihood per iteration is used to assess the stability of the log-likelihood during the sampling iterations. **D-F.** cisTopic modelling using the summits as called by MACS extended +/- 250 bp as regulatory regions (16,417 regions). The log-likelihood per model, the log-likelihood per iteration for selected models, and the cisTopic cell tSNE of the selected model (with 49 topics) are shown. **G-H.** cisTopic modelling using the cisTarget regions as regulatory regions (129,553 regions). The log-likelihood per model, the log-likelihood per iteration for selected models, and the cisTopic cell tSNE of the selected model (with 49 topics) are shown.

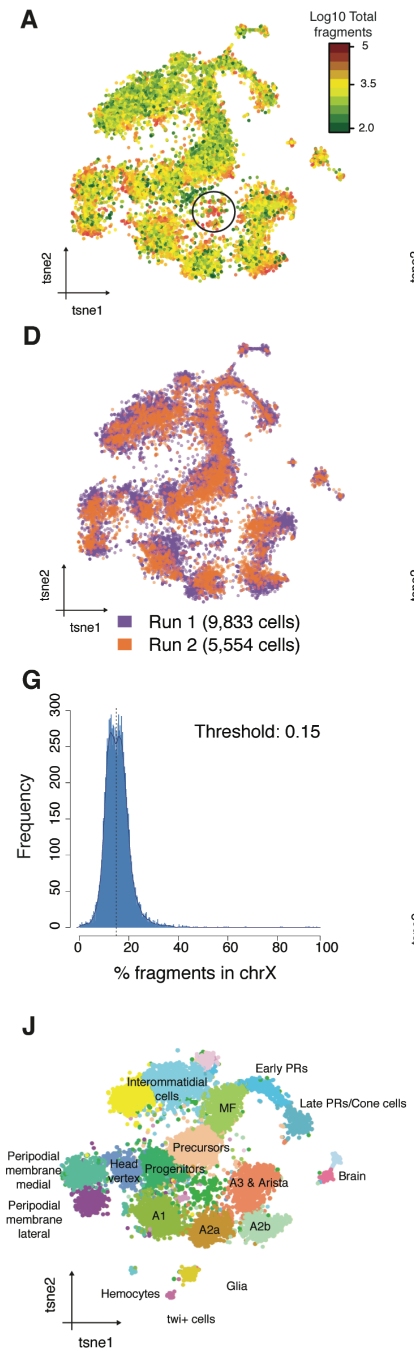

Figure S8. Cell clustering is driven by cell type and not by batch effects. A. cisTopic cell tSNE (15,387 cells) colored by normalized number of fragments ($\log_{10}$). **B.** cisTopic cell tSNE colored by normalized number of accessible regions ($\log_{10}$) **C.** cisTopic cell tSNE colored by percentage of fragments that overlap bulk peaks. **D.** cisTopic cell tSNE colored by experimental run. **E.** cisTopic cell tSNE colored by topic 46 enrichment. A representative enriched motif with Normalised Enrichment Score (NES) is shown. **F.** cisTopic cell tSNE colored by topic 18 enrichment. Representative enriched motifs with NES are shown. **G.** Histogram showing the bimodal distribution of fragments over the X chromosome. Cells with more than 15% of fragments on the X chromosome are classified as females;

otherwise, as males. **H.** cisTopic cell tSNE colored by assigned sex. **I.** cisTopic cell tSNE colored by topic 4 enrichment. A representative enriched motif with NES is shown. **J.** cisTopic cell tSNE after removing the run and the sex-specific topics.

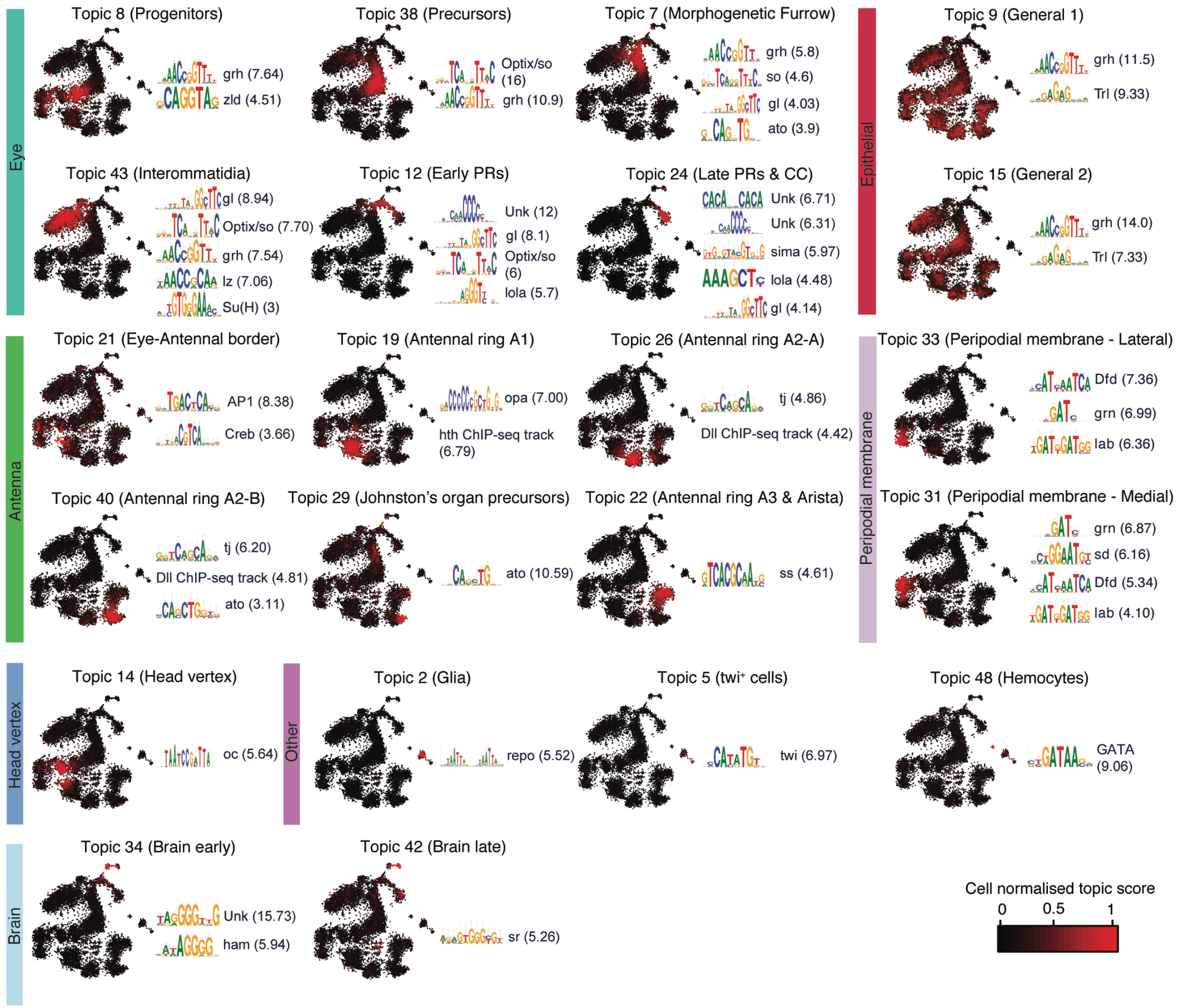

Figure S9. Motif enrichment on the regions within regulatory topics reveals key master regulators of the cell populations in the eye-antennal disc. For each selected topic, cisTopic cell tSNE colored by topic enrichment accompanied by representative enriched motifs (which are linked to known master regulators of each cell type) with Normalized Enrichment Score (NES).

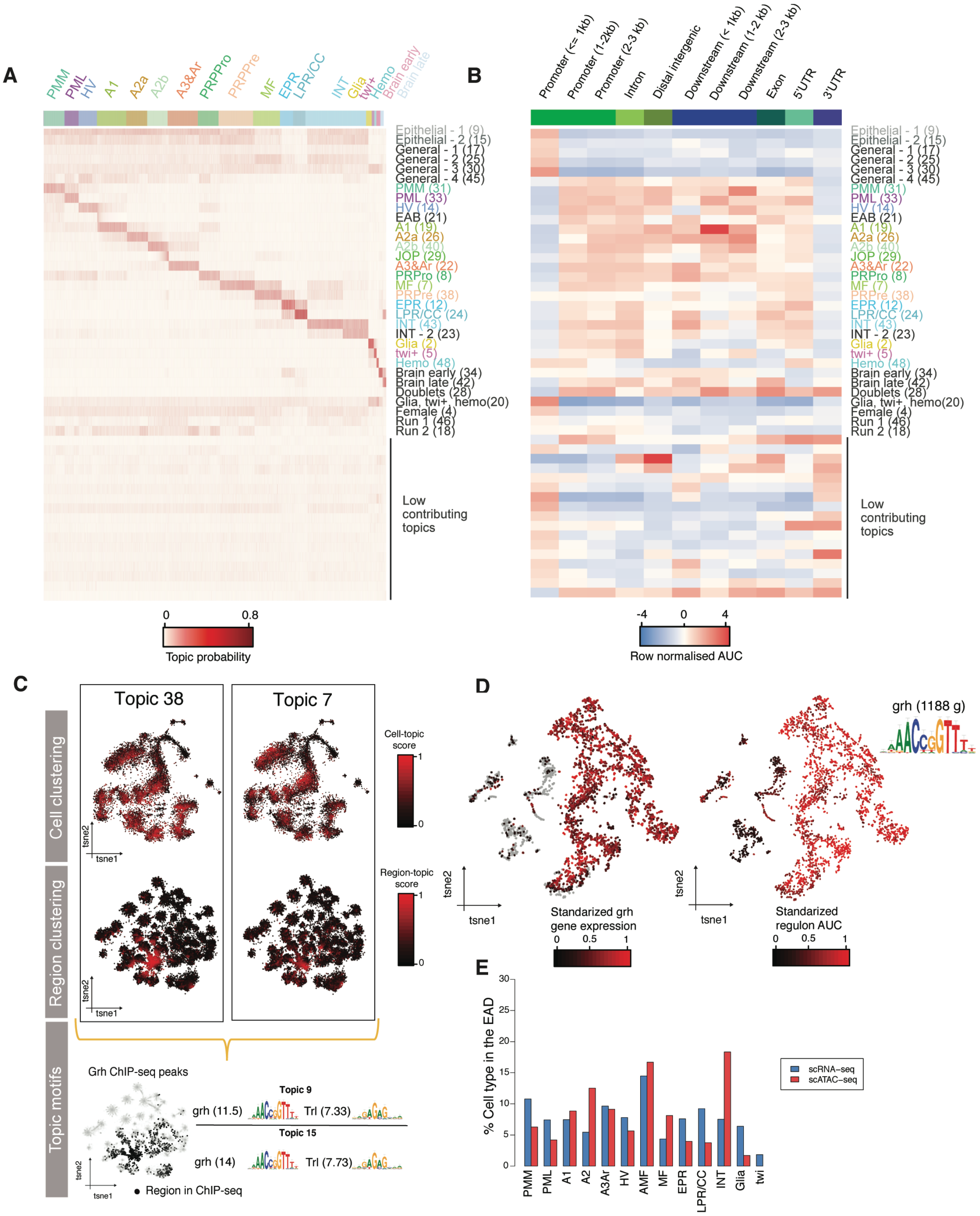

Figure S10. Annotation of topics and cell clusters from scATAC-seq profiles. A. cisTopic cell-topic heatmap showing the probability of each topic in each cell and the topic annotation. **B.** Heatmap showing the enrichment of each regions class in each topic. **C.** Epithelial topics are enriched on Grh binding sites. Top: cisTopic cell tSNE colored by topic enrichment. Middle: cisTopic region tSNE colored by topic enrichment. Bottom: cistopic region tSNE colored by Grh ChIP-seq peaks, together with representative motifs enriched in these topics with their associated TF and cisTarget Normalized Enrichment Score (NES). **D.** Seurat scRNA-seq tSNE colored by grh gene expression (top) and grh

regulon enrichment (bottom). **E.** Proportion of cell types in the eye-antennal disc based on scRNA-seq and scATAC-seq analysis. Ar: Arista. EPR: Early photoreceptors. Hemo: Hemocytes. HV: Head Vertex. INT: Interommatidial cells. JOP: Johnston Organ Precursor. LPR/CC: Late photoreceptors and cone cells. MF: Morphogenetic Furrow. PML: Peripodial Membrane Lateral. PMM: Peripodial Membrane Medial. PRPre: Photoreceptor precursors. PRPro: Photoreceptor progenitors.

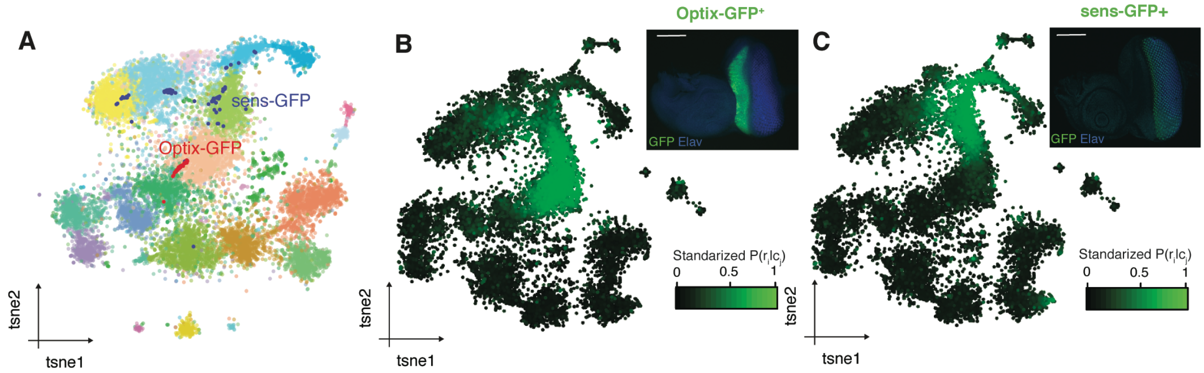

Figure S11. Validation of cell type annotations using previously published FAC sorted single-cell ATAC-seq data. A. cisTopic cell tSNE after the integration of FAC-sorted cells profiled by Fluidigm C1. Cells profiled with 10X are shown with transparency colored by annotation. **B.** cisTopic cell tSNE colored by the probability of the region corresponding to the Optix2/3 enhancer, whose activity is measured by GFP signal in the adjacent image. Scale bar: 100um. **C.** cisTopic cell tSNE colored by the probability of the region corresponding to the sens-F2 enhancer, whose activity is measured by GFP signal in the adjacent image. Scale bar: 100um.

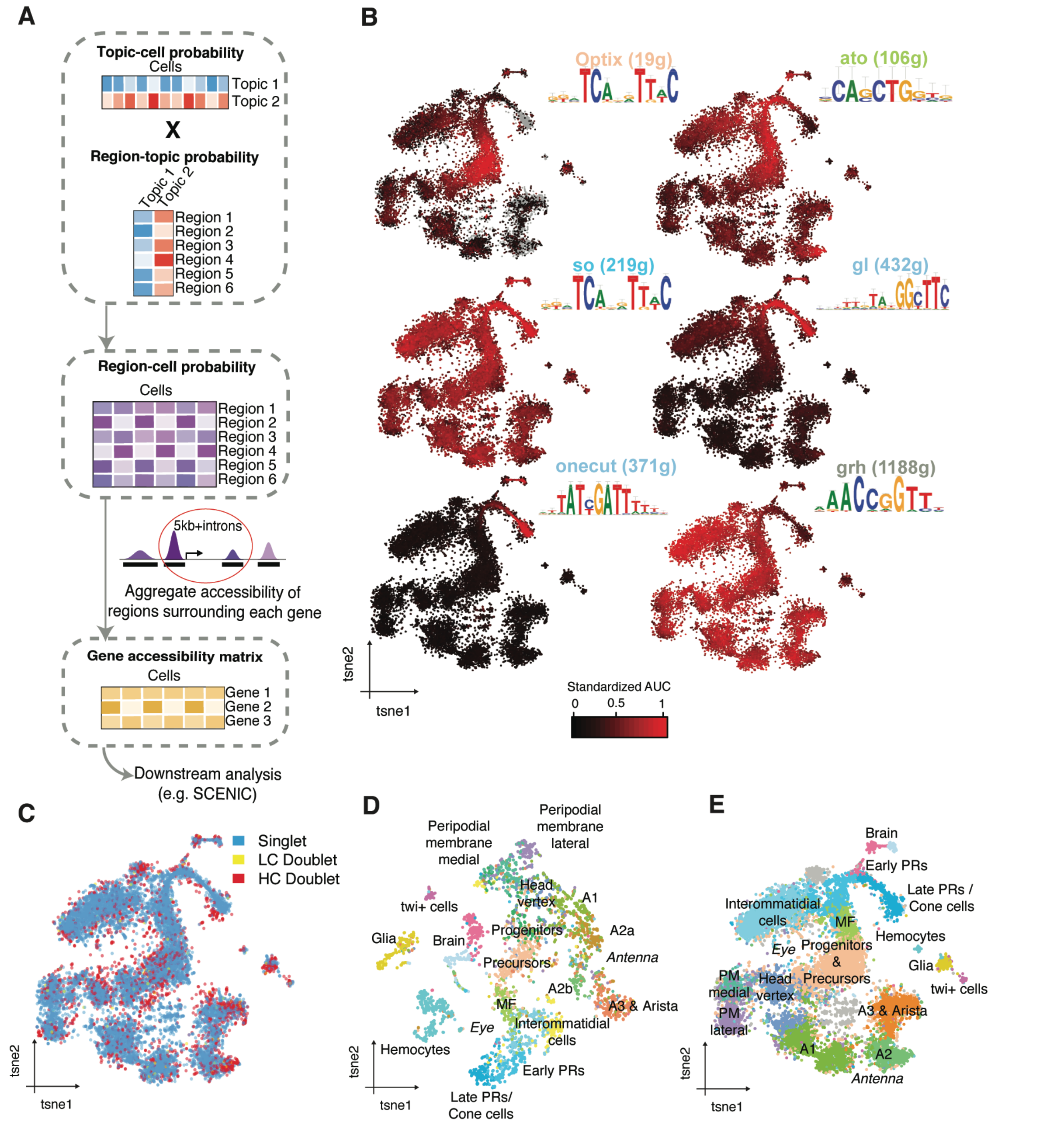

Figure S12. Derivation of gene activity scores using scATAC-seq data and comparsion with gene expression. A. cisTopic topic-cell and region-topic distributions can be exploited to predict the probability of each region in each cell. By aggregating the probabilities of regions around the TSS of each gene (in this case, 5kb upstream and introns), a gene accessibility matrix can be derived. **B.** cisTopic cell tSNE is colored based on the enrichment of regulons derived from scRNA-seq, evaluated in the gene accessibility matrix. C**.** Annotation of singlets and doublets based on DoubletFinder (on the gene accessibility matrix). **D.** Seurat scRNA-seq tSNE colored by the labels transferred from the scATAC-seq annotation. **E.** cisTopic cell tSNE colored by the scRNA-seq annotation after label transferring with Seurat v3 using cisTopic gene accessibility matrix.

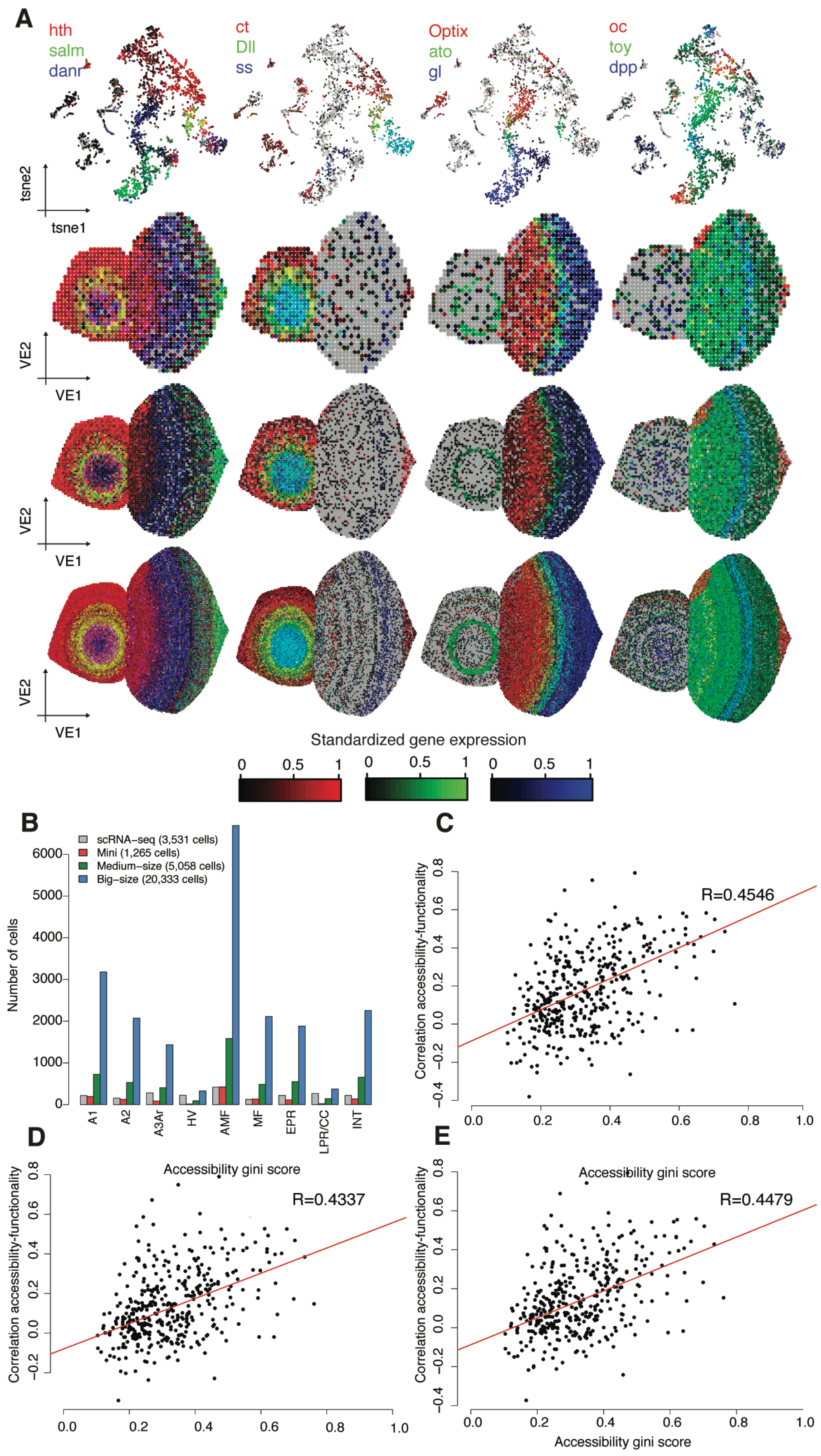

Figure S13. Assessment of the size of the virtual template. A. Representation of Figure 3B using templates of different sizes. Top: Seurat tSNE (3,531 cells) colored by gene expression. Middle top: Mini virtual eye (1,265 cells) colored by gene expression. Middle bottom: Medium-size virtual eye (5,058 cells) colored by gene expression. Bottom: Big-size virtual eye (20,333 cells) colored by gene

expression. B. Number of cells per cell type in the original scRNA-seq data and on the different virtual templates. **C.** Relationship between the correlation between the accessibility and the activity of the regions and their distribution (as gini score) using the mini virtual eye. **D.** Relationship between the correlation between the accessibility and the activity of the regions and their distribution (as gini score) using the medium-size virtual eye. **E**. Relationship between the correlation between the accessibility and the activity of the regions and their distribution (as gini score) using the big-size virtual eye. We selected the initial size of the virtual eye (with 5,058 cells) to sample as many single-cell ATAC-seq profiles (~15K cells) without over-sampling single-cell RNA-seq profiles (~3.5K). Indeed, in the medium-size virtual eye, each cell is sampled a median of 1.07 times (mean: 1.6); while in the mini virtual eye, each cell is sampled a median of 0.42 (mean: 0.5; meaning that not all profiles are exploited); and in the big-size virtual eye each cell is sampled a median of 4 times (mean 6.4; meaning that profiles are re-used several time).

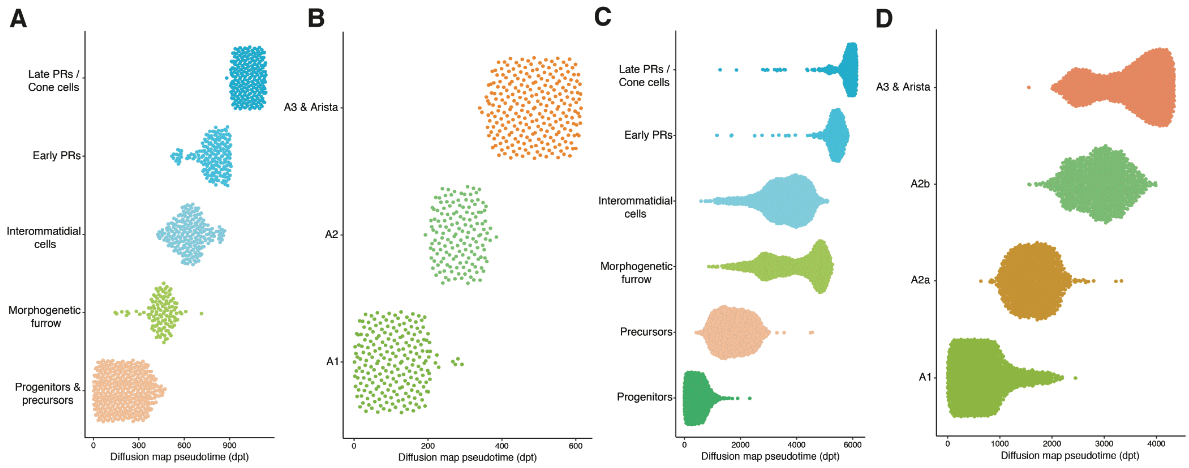

Figure S14. Ordering cell types in the eye and antenna by pseudotime. A. Eye cell types ordered by pseudotime using scRNA-seq data. **B**. Antennal cell types ordered by pseudotime using scRNA-seq data. **C.** Eye cell types ordered by pseudotime using scATAC-seq data. **D.** Antennal cell types ordered by pseudotime using scATAC-seq data.

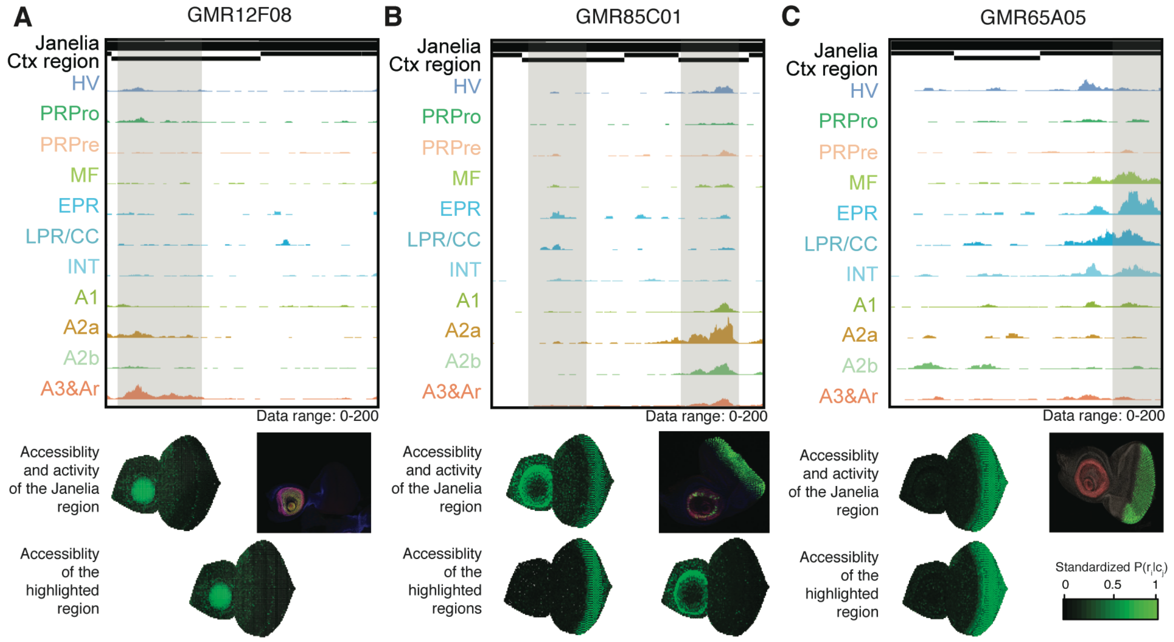

Figure S15. Wide Janelia regions contain multiple enhancers. A. Example of a Janelia region containing one enhancer (highlighted). **B.** Example of a Janelia region containing two enhancers. The activity of the Janelia region is the result of both enhancers (highlighted). **C**. Example of a Janelia region with multiple enhancers. The activity of the Janelia image is likely the result of the highlighted regions, but not the others. Aggregate profiles are shown on top, the accessibility of the Janelia region in the virtual cells (calculated by the aggregation of the region-cell probabilities of the regions within the Janelia region) and its activity, together with the accessibility of the highlighted regions, are shown below. Janelia enhancer activity images are taken from the Janelia Flylight project (Jory et al., 2012). Ar: Arista. EPR: Early photoreceptors. HV: Head Vertex. INT: Interommatidial cells. LPR/CC: Late photoreceptors and cone cells. MF: Morphogenetic Furrow. PRPre: Photoreceptor precursors. PRPro: Photoreceptor progenitors.

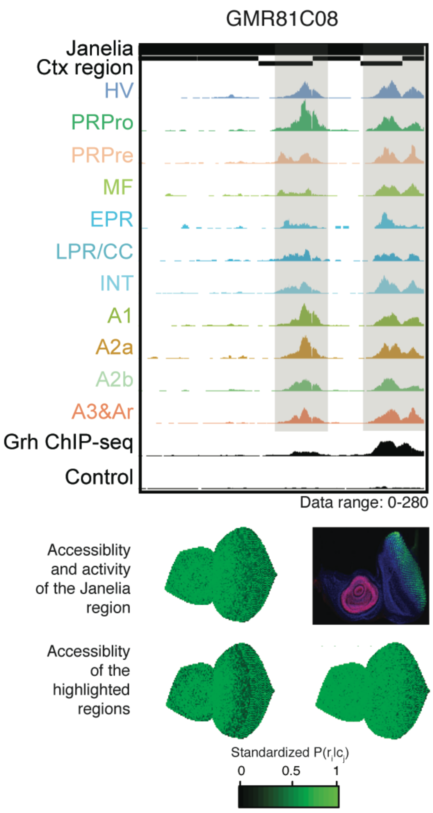

Figure S16. Janelia regions lowly correlated with activity correspond to primed enhancers. Example of a Janelia region with negative correlation between accessibility and activity (with correlation: -0.34). Aggregate profiles and the Grh Chip-seq profile are shown on top, while the accessibility of the Janelia region in the virtual cells (calculated by the aggregation of the region-cell probabilities of the regions within the Janelia region) and its activity, together with the accessibility of the highlighted regions, are shown below. The Janelia enhancer activity image is taken from the Janelia Flylight project (Jory et al., 2012). Ar: Arista. EPR: Early photoreceptors. HV: Head Vertex. INT: Interommatidial cells. LPR/CC: Late photoreceptors and cone cells. MF: Morphogenetic Furrow. PRPre: Photoreceptor precursors. PRPro: Photoreceptor progenitors.

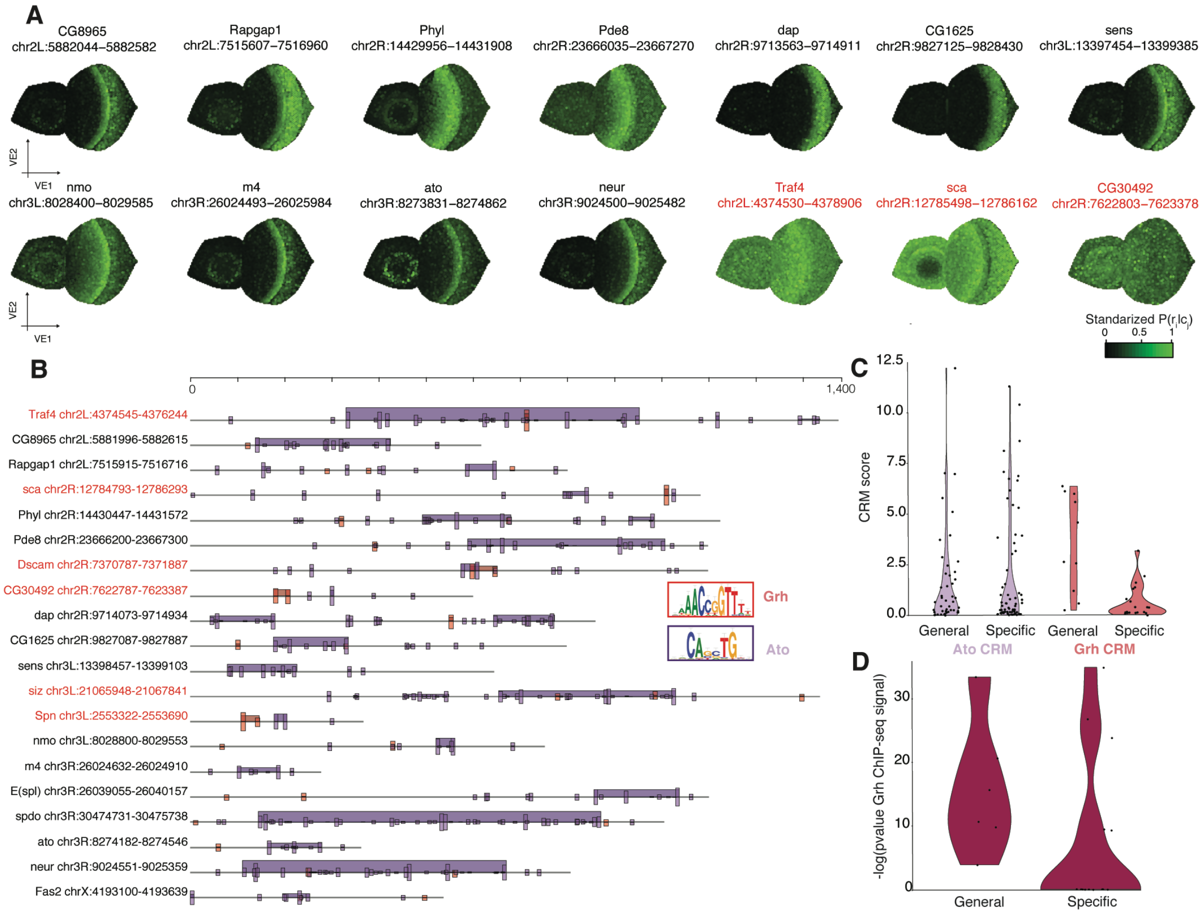

Figure S17. Uncoupling enhancer accessibility and activity in validated atonal enhancers from Aerts *et al.* **a.** Virtual eye-antennal disc colored by the accessibility probability of 14 of the atonal enhancers. Enhancers with general accessibility and enriched for grh binding sites are shown in red. **b.** TOUCAN view showing the location of atonal (purple) and grh (red) motifs and Cis-Regulatory Modules (CRMs). Enhancers with general accessibility and enriched for grh binding sites are shown in red. **c.** Violin plot comparing CRMs scores in specific and generally accessible regions. **d.** Violin plot comparing MACS2 log10(p-val) of the grh ChIP-seq peaks in specific and generally accessible regions. Jacobs *et al.*(Jacobs et al., 2018) showed that Grh and Ato enhancers had a higher ATAC-seq signal compared to uniquely Ato targets. Here we find that these differences in signal are due to Ato enhancers being uniquely accessible in the morphogenetic furrow, while shared Grh and Ato enhancers are accessible across all epithelial cells.

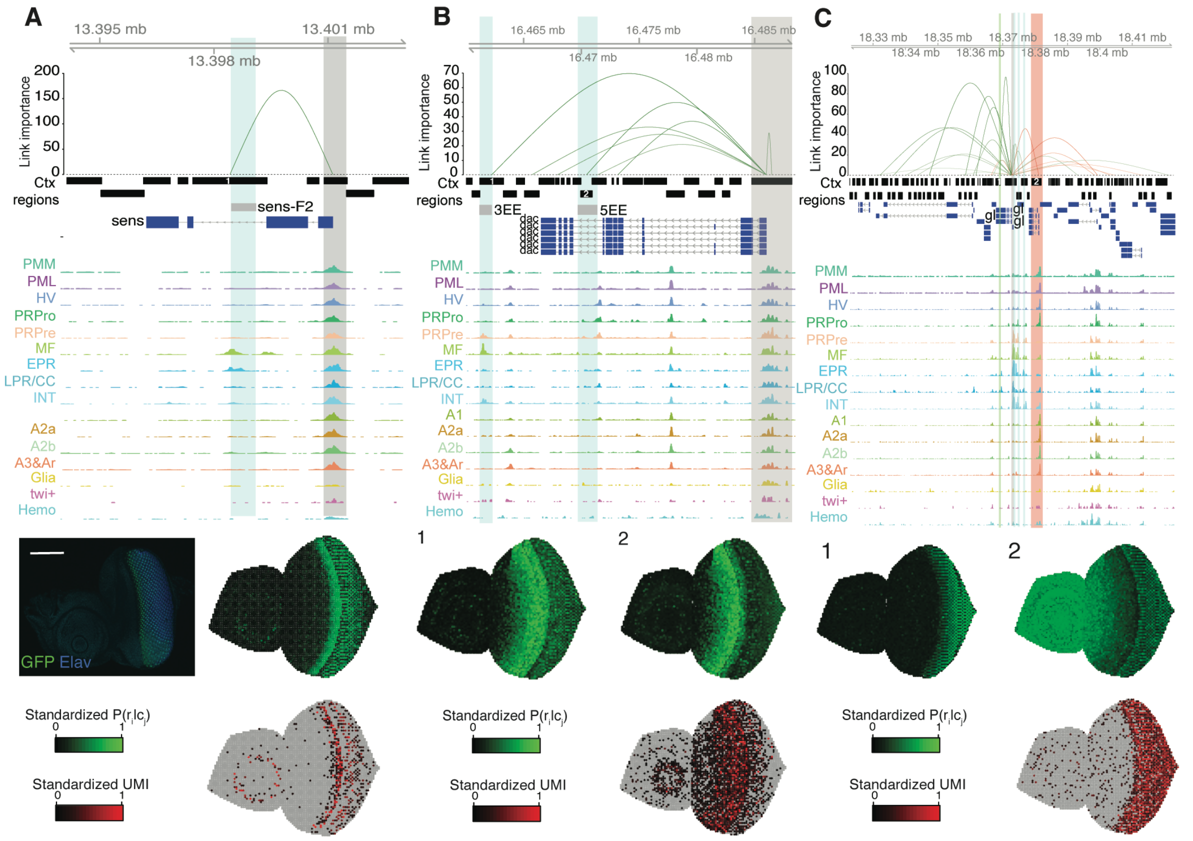

Figure S18. Enhancer-to-target links support basic principles of gene regulation. A. Example of a gene (*sens*) controlled by uniquely one enhancer (sens-F2). Linkage importance is shown on top, followed by the cisTarget regions, the sens-F2 enhancer, gene annotation and the aggregated ATAC-seq profiles of the different cell types. The activity of sens-F2 is shown below, together with the virtual eye-antennal disc colored by *sens* gene expression (red) and sens-F2 accessibility probability (green). Scale bar: 100 µm. **B.** Enhancer-to-target links for the *dac* gene. From top to bottom are shown: position, link importance, cisTarget regions, validated *dac* enhancers, gene annotation and scATAC-seq aggregates by cell type. The expression of *dac* is shown on the virtual eye in a red scale, the accessibility probability of 3EE (1) and 5EE (2) is shown in a green scale. The validated enhancers are highlighted in blue, and the promoter of *dac* is highlighted in grey. **C.** Enhancer-to-target links for the *gl* gene. From top to bottom are shown: position, link importance, cisTarget regions, gene annotation and scATAC-seq aggregates by cell type. The expression of *gl* is shown on the virtual eye in a red scale, the accessibility probability of an activating and a repressing enhancer (highlighted in green and red, respectively) is shown in a green scale. Validated *gl* enhancers are highlighted in blue, and the promoter of *gl* is highlighted in grey. Ar: Arista. EPR: Early photoreceptors. Hemo: Hemocytes. HV: Head Vertex. INT: Interommatidial cells. JOP: Johnston Organ Precursor. LPR/CC: Late photoreceptors and cone cells. MF: Morphogenetic Furrow. PML: Peripodial Membrane Lateral. PMM: Peripodial Membrane Medial. PRPre: Photoreceptor precursors. PRPro: Photoreceptor progenitors.

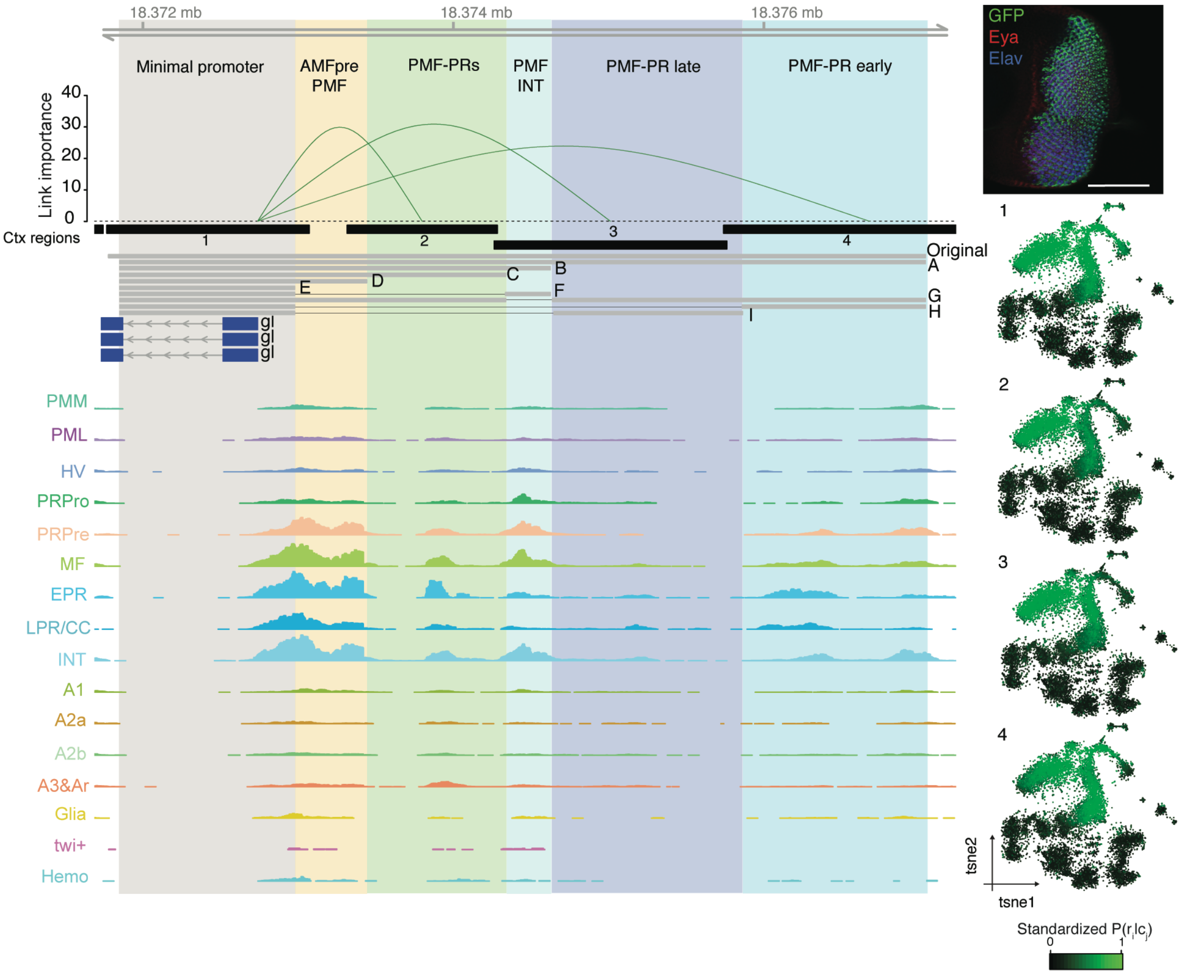

Figure S19. Gene expression is regulated by complex combinations of enhancers. Example of a gene (gl) controlled by multiple enhancers, validated by Fritsch *et al.* (Fritsch et al., 2019). Linkage importance is shown on top, followed by the cisTarget regions, the constructs tested by the authors, gene annotation and the aggregated ATAC-seq profiles of the different cell types. Highlighted areas indicate the cell types in which those segments of the sequence result in activity. The activity of the Glass Multimer Reporter (GMR) is shown, together with the cisTopic cell tSNE colored by the accessibility of the marked regions. Ar: Arista. EPR: Early photoreceptors. Hemo: Hemocytes. HV: Head Vertex. INT: Interommatidial cells. JOP: Johnston Organ Precursor. LPR/CC: Late photoreceptors and cone cells. MF: Morphogenetic Furrow. PML: Peripodial Membrane Lateral. PMM: Peripodial Membrane Medial. PRPre: Photoreceptor precursors. PRPro: Photoreceptor progenitors.

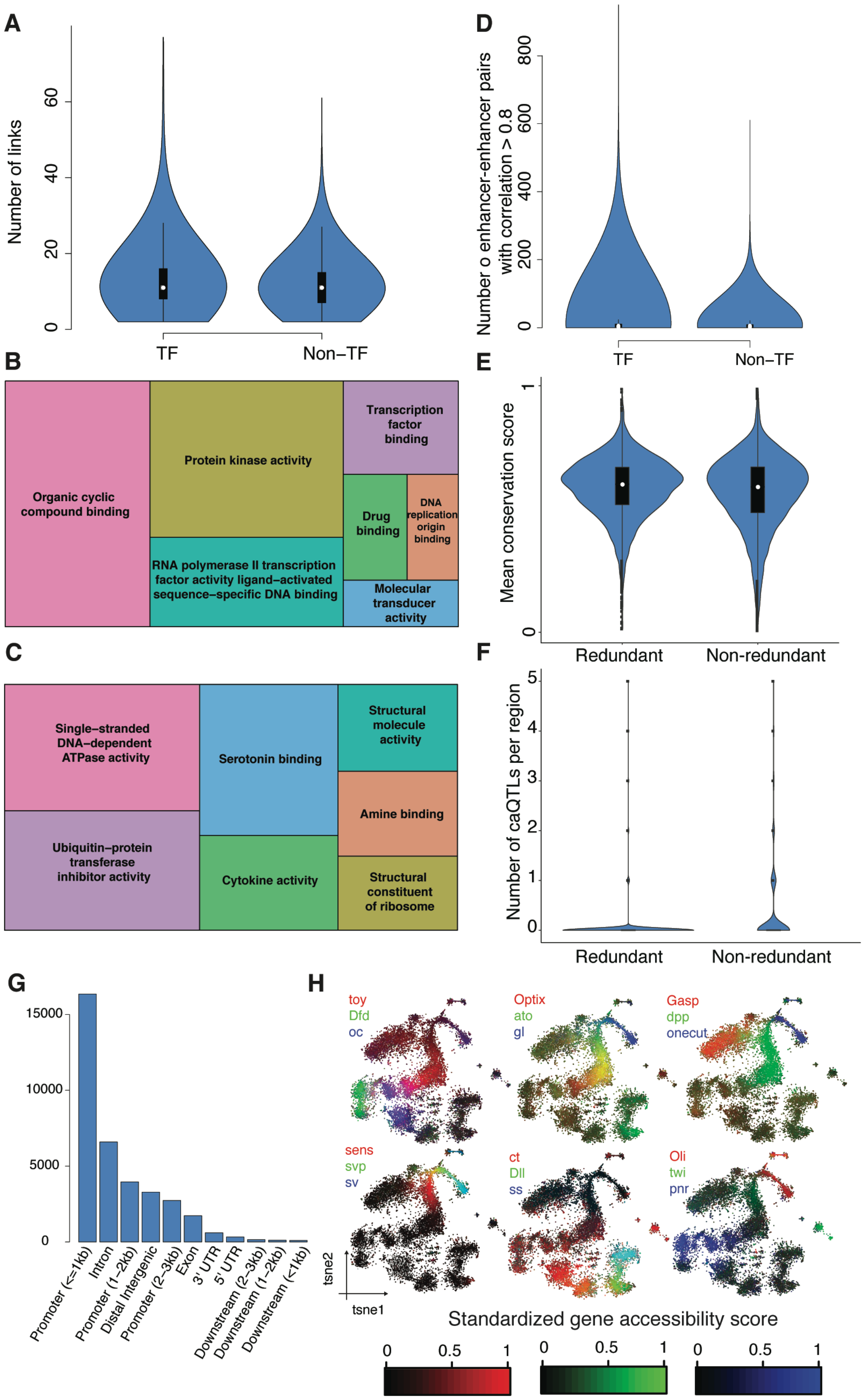

Fig S20. Enhancer-to-target genes recapitulate key aspects of gene regulation. A. Number of links per TF and non-TF gene. **B.** Revigo view of the functional GO terms enriched found by GOrilla in the genes with a high number of links (based on the decreasing ranking by number of genes of the links).

C. Revigo view of the functional GO terms enriched found by GOrilla in the genes with a low number of links (based on the ascending ranking by number of genes of the links). **D.** Number of enhancer-enhancer pairs with a correlation above 0.8 (based on the accessibility probability) for TF and non-TF genes. **E.** Mean conservation score (PhastCons) for redundant and non-redundant enhancers. H_0: Average conservation score on redundant enhancers (0.585) <= Average conservation score on non-redundant enhancers (0.567); p-value: 1.2×10^{-13}. **F.** Number of caQTLs per region for redundant and non-redundant enhancers. H_0: Number of caQTLs on redundant enhancers (mean: 0.12) >= Number of caQTLs on non-redundant enhancers (mean:0.20); Wilcoxon rank sum test p-value: 2.48×10^{-5}. **G.** Classification of the regions involved in the links. **H.** Predicted gene expression (or gene accessibility) based on the signed aggregation of the probabilities of the enhancers linked to each gene weighted by importance. Three genes are shown per plot, using RGB encoding.

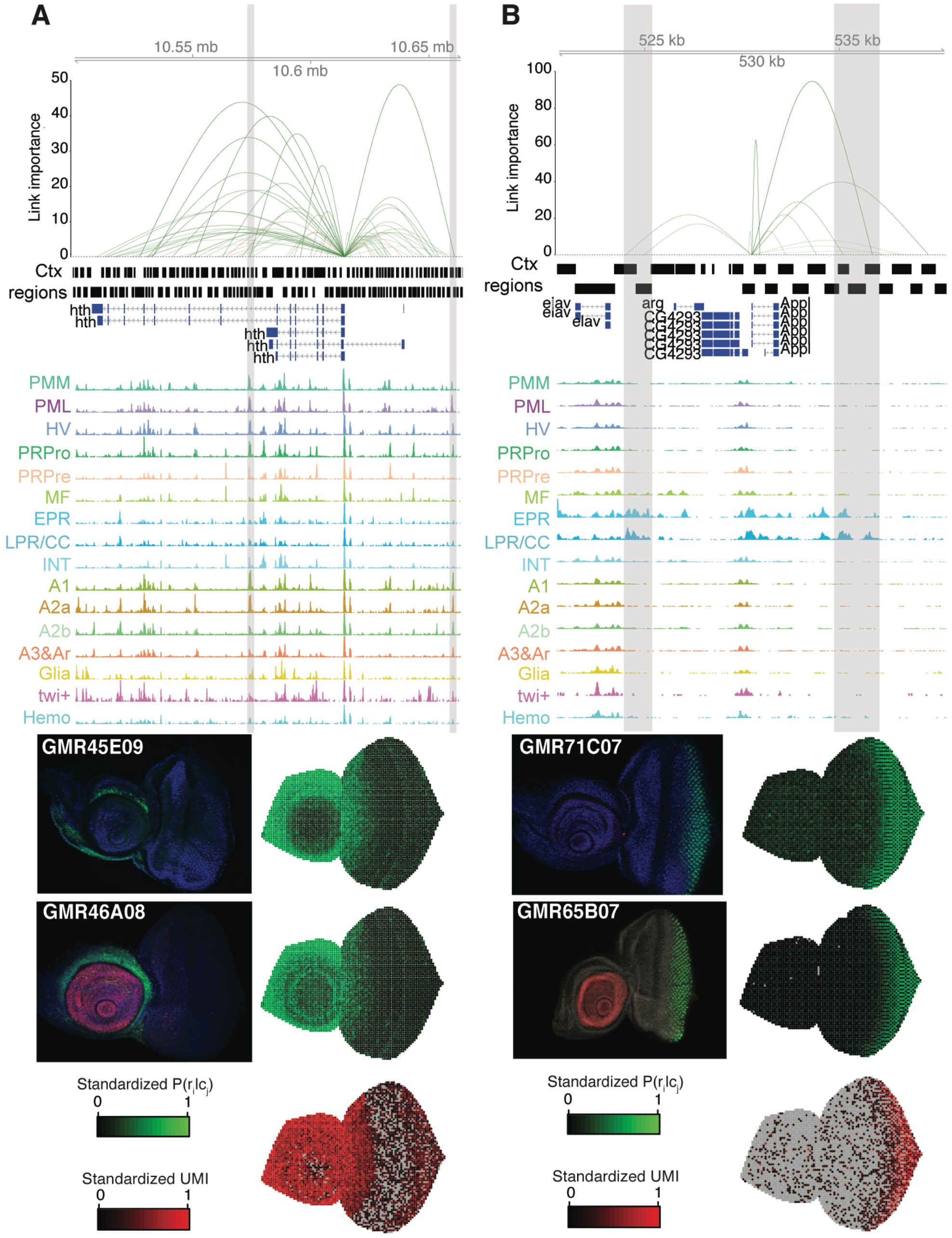

Figure S21. Genes are regulated by many enhancers, some of which show redundant activity (and accessibility). Enhancer-to-target links for hth (a) and Appl. Top: Links between enhancer and target genes, whose height represent their importance. Midde top: cisTarget regions. Middle: Genome annotation. Middle bottom: Normalized aggregate ATAC-seq profiles per cell type. Bottom: Enhancer activity (images taken from the Janelia Flylight Project), and virtual eye colored by the predicted enhancer accessibility (green) and the expression of the gene (red). Redundant enhancers are highlighted in grey. Ar: Arista. EPR: Early photoreceptors. Hemo: Hemocytes. HV: Head Vertex. INT: Interommatidial cells. JOP: Johnston Organ Precursor. LPR/CC: Late photoreceptors and cone cells. MF: Morphogenetic Furrow. PML: Peripodial Membrane Lateral. PMM: Peripodial Membrane Medial. PRPre: Photoreceptor precursors. PRPro: Photoreceptor progenitors.

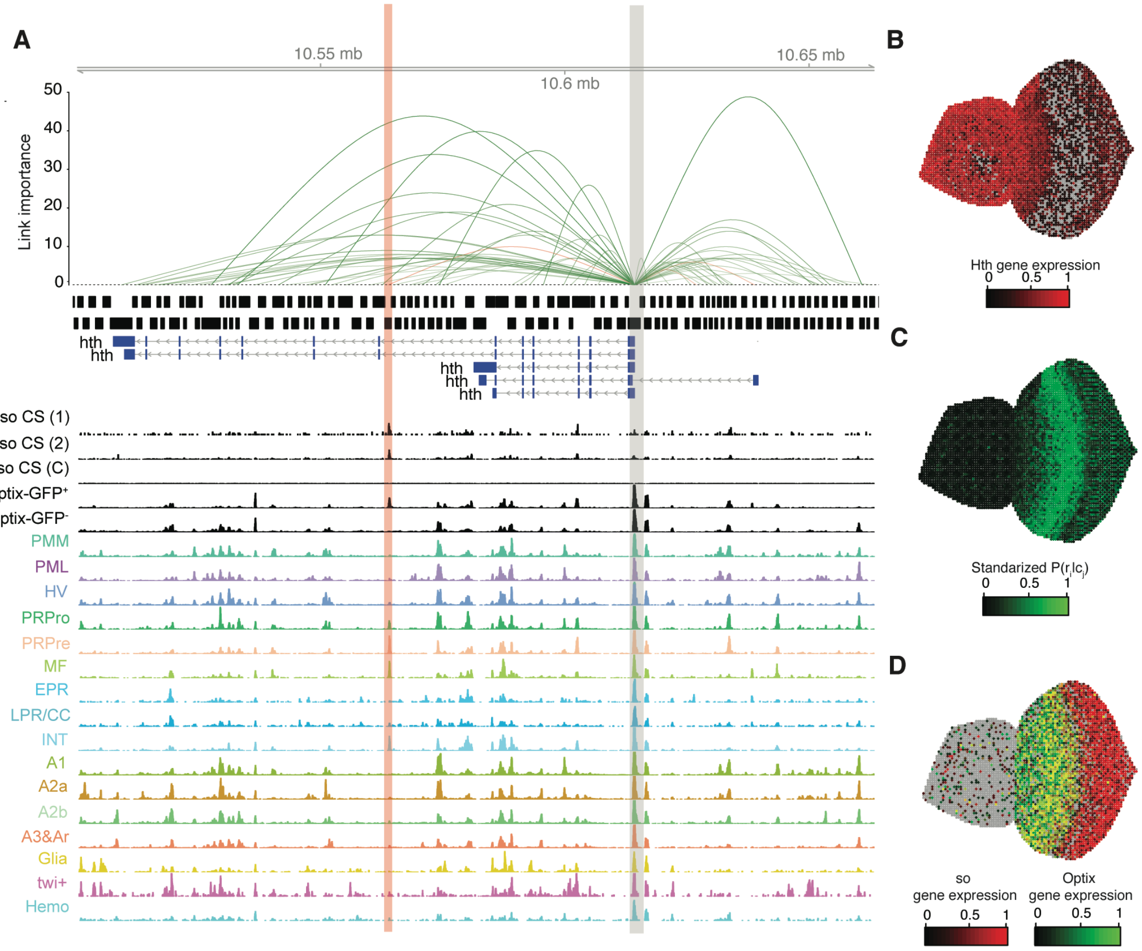

Figure S22. Hth is potentially repressed by an enhancer bound by so and also accessible in FAC-sorted Optix-GFP$^+$ cells. a. Enhancer-to-target links for the hth gene. From top to bottom are shown: position, link importance, cisTarget regions, gene annotation, so ChIP-seq data (replicate 1, replicate 2 and control), Optix-GFP$^+$ cells bulk ATAC-seq profile, Optix-GFP$^-$ cells bulk ATAC-seq profile and scATAC-seq aggregates by cell type. The repressive enhancer is highlighted in red, the promoter of hth is highlighted in grey. **b.** Virtual eye-antennal disc colored by the standarized gene expression of hth. **c.** Virtual eye-antennal disc colored by the standarized accessibility probability of the represive enhancer. **d.** Virtual eye-antennal disc colored by the standarized gene expression of so (red) and Optix (green). Ar: Arista. EPR: Early photoreceptors. Hemo: Hemocytes. HV: Head Vertex. INT: Interommatidial cells. JOP: Johnston Organ Precursor. LPR/CC: Late photoreceptors and cone cells. MF: Morphogenetic Furrow. PML: Peripodial Membrane Lateral. PMM: Peripodial Membrane Medial. PRPre: Photoreceptor precursors. PRPro: Photoreceptor progenitors.

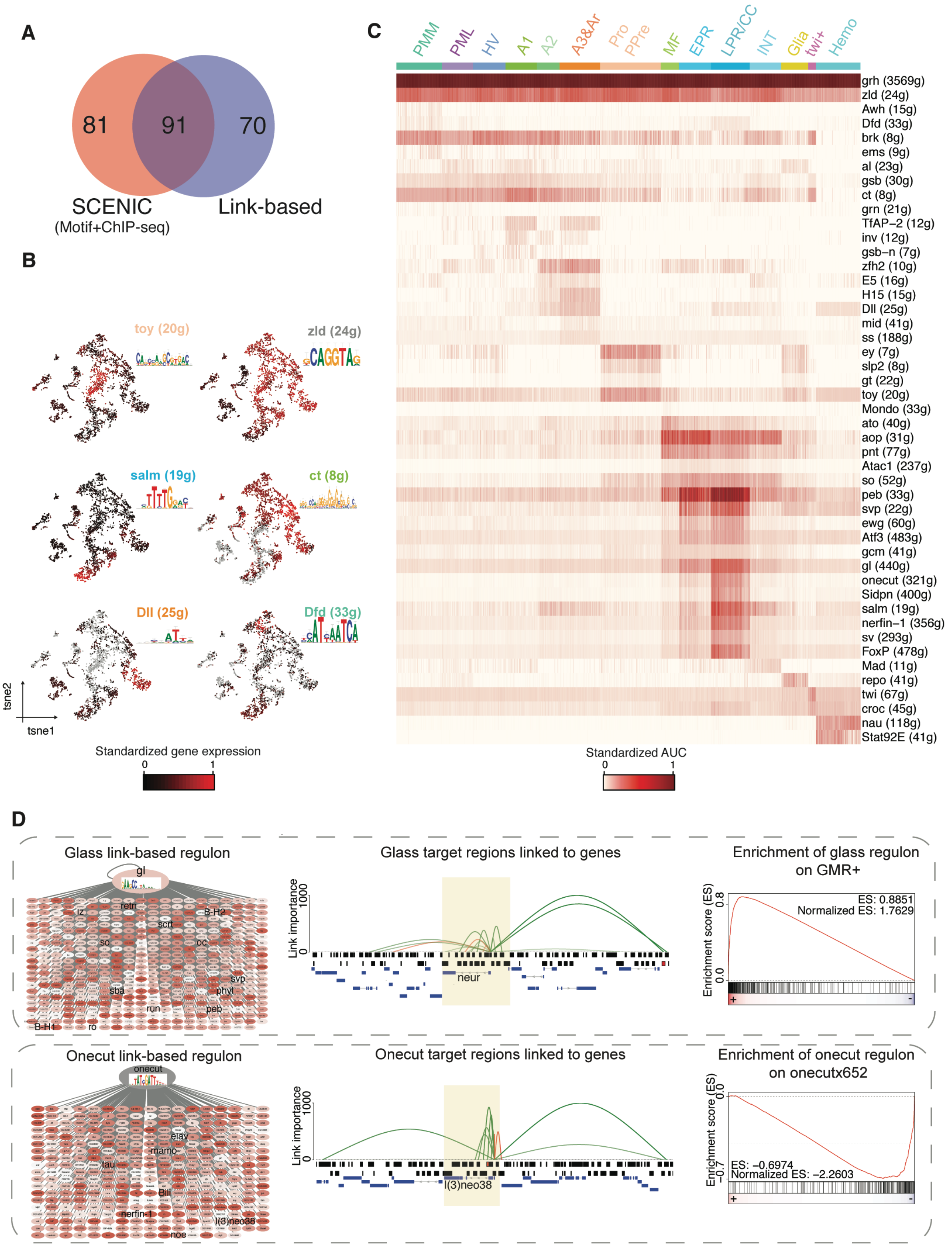

Figure S23. Link-based regulons identify additional master regulators during the eye-antennal disc development. A. Venn diagram showing the overlap between SCENIC and link-based regulons. **B.** Seurat scRNA-seq tSNE colored by regulon enrichment in each cell. **C.** Cell-to-regulon heatmap showing the standardized enrichment or Area Under the Curve (AUC) from SCENIC for each selected

regulon based on RSS in each cell. **D.** Link-based regulons for Glass and Onecut, built using GRNBoost co-expression modules and motif enrichment on the regions linked to each potential target gene. Left: Cytoscape view of the link-based regulons. Color scale indicates the average importance of the regions enriched in the transcription factor motif for each gene. For the Glass regulon, known target genes (Potier et al., 2014) are highlighted; for the Onecut regulon, representative genes involved in neuronal differentiation are highlighted. Middle: Examples of target genes, showing the enhancer-to-region links (top), cisTarget regions (middle) and gene annotation. cisTarget regions in which the motif for the transcription factor is enriched are shown in red. The area highlighted in yellow corresponds to the motif enrichment search space used in SCENIC (Aibar et al., 2017). Right: GSEA plots comparing the link-based regulons with differentially expressed genes in a compendium of conditions compared to wild type. We score the gl regulon, against GMR^{+} FAC sorted cells from Potier *et al.*; and the onecut regulon, against a loss-of-function mutant of onecut, also presented by Potier *et al.*(Potier et al., 2014). Ar: Arista. EPR: Early photoreceptors. Hemo: Hemocytes. HV: Head Vertex. INT: Interommatidial cells. JOP: Johnston Organ Precursor. LPR/CC: Late photoreceptors and cone cells. MF: Morphogenetic Furrow. PML: Peripodial Membrane Lateral. PMM: Peripodial Membrane Medial. PRPre: Photoreceptor precursors. PRPro: Photoreceptor progenitors.

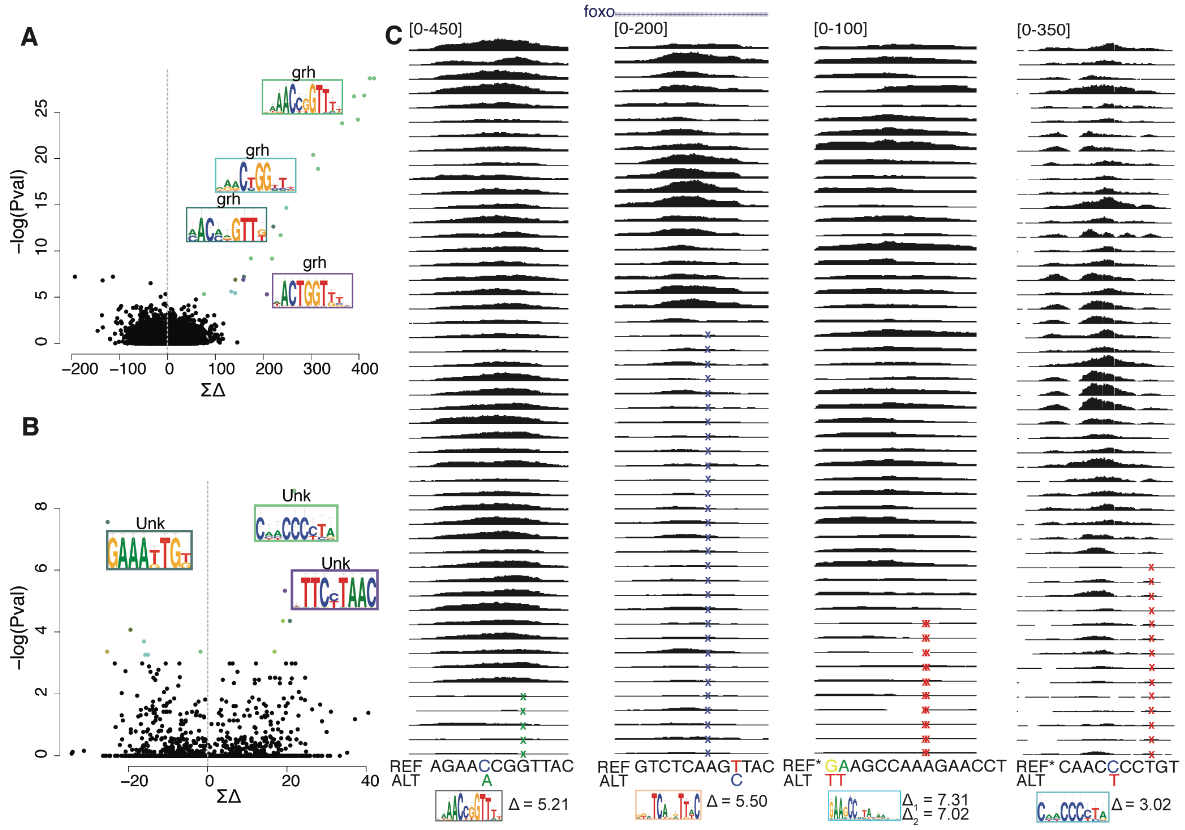

Figure S24. Cell-type specific analysis of chromatin accessibility QTLs reveals additional motifs involved chromatin opening compared to bulk analysis. a. Delta motif plot, showing the cumulative effect of the 10,969 caQTLs found genome-wide on the CRM score of 24,454 motifs on the x-axis. The y-axis shows the enrichment of motifs affected by caQTLs compared to control SNPs genome-wide (one-sided Fisher's exact test, log transformed). **b.** Delta motif plot, showing the cumulative effect of the 323 caQTLs found on regions specifically accessible in late PRs and cone cells (Topic 24) on the CRM score of 24,454 motifs on the x-axis. The y-axis shows the enrichment of motifs affected by caQTLs compared to controls SNPs (one-sided Fisher's exact test, log transformed). **c.** Bulk ATAC-seq profiles of the inbred lines, on regions affected by caQTLs that modify the highlighted motifs. The caQTLs coordinates are, from left to right: chr3L:17392596, chr3R:14076593, chr2R:18674001 and chr2R:18674002, and chr3R:29376820. REF sequences with an * indicate that the reverse complement strand is given.

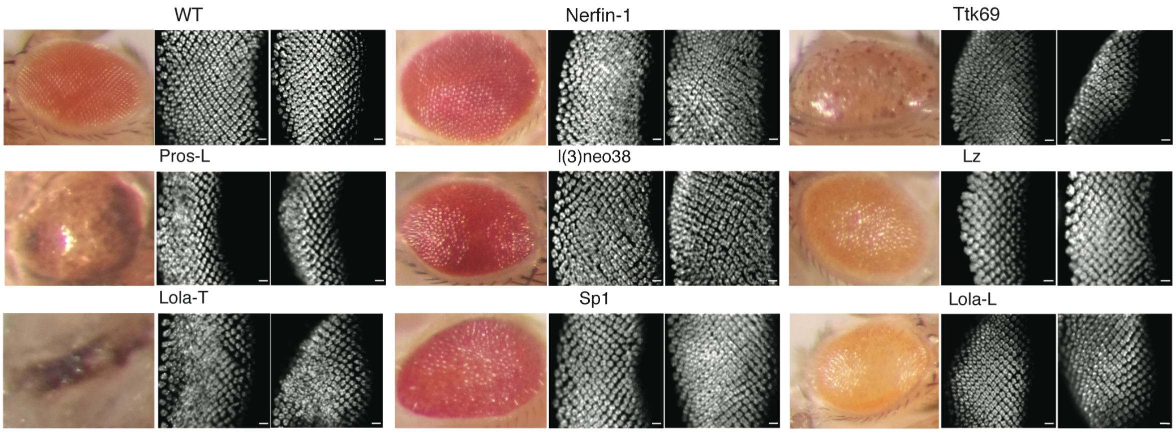

Figure S25. Overexpression of 13 TFs using the GMR-GAL4/UAS-TF system reveals 8 master regulators whose overexpression causes defects during the development of the eye disc. The adult eye in each of these lines is shown (except for Pros and Lola-T), together with the elav staining on posterior to the morphogenetic furrow in the third instar larvae eye disc on two biological replicates. Scale bar: 10 um.

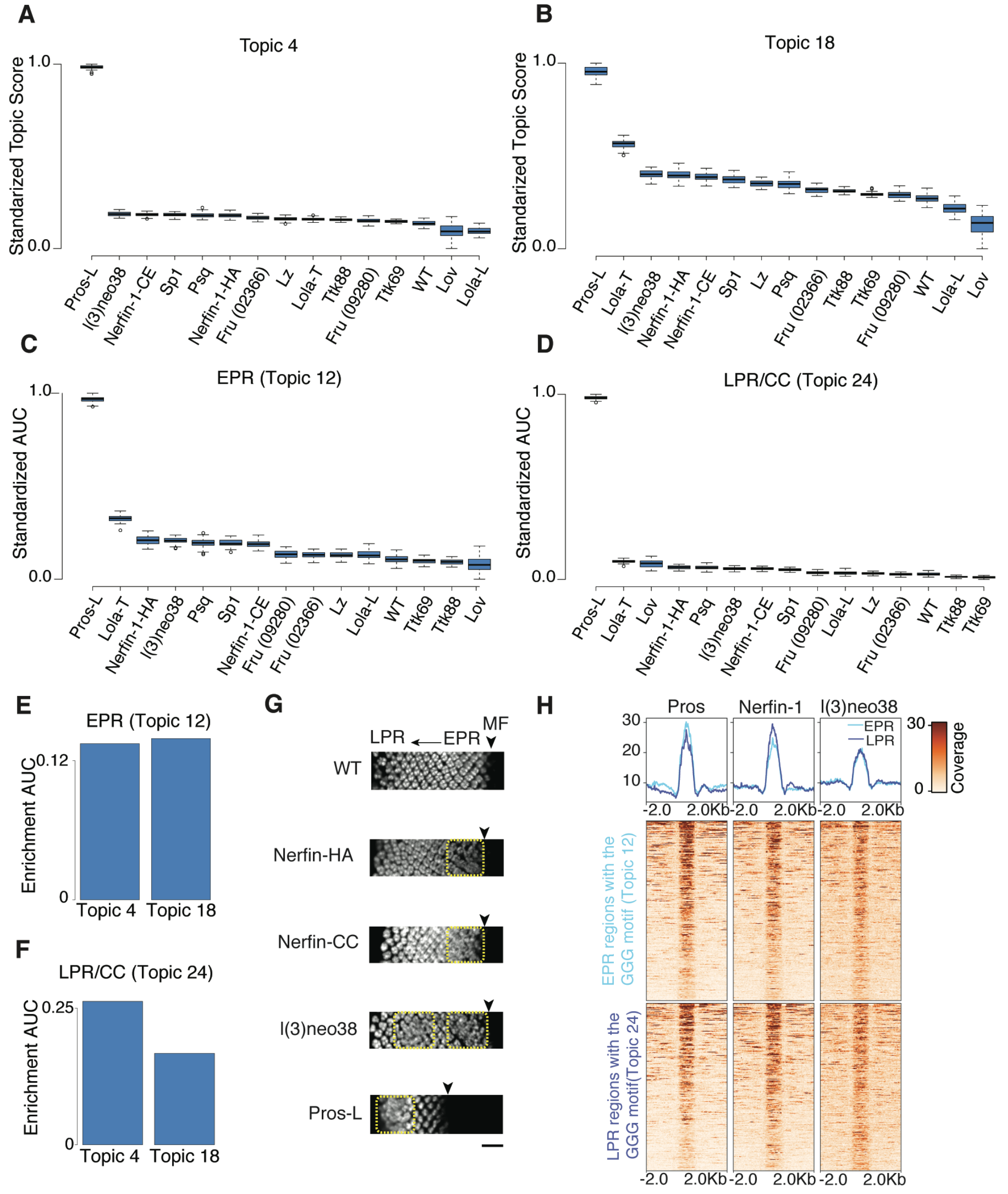

Figure S26. Pros-L overexpression result on the opening of GGG enriched regions. a. Standarized topic 4 enrichment (from the bootstrapped analysis) on the bootstrapped samples. **b.** Standarized topic 18 enrichment (from the bootstrapped analysis) on the bootstrapped samples. **c.** Standarized enrichment of the early photoreceptor regions (topic 12) on the bootstrapped samples. **d.** Standarized enrichment of the late photoreceptor regions (topic 24) on the bootstrapped samples. **e.** AUC enrichment of the early photoreceptor regions on the topics 4 and 18 (from the bootstrapped analysis). **f.** AUC enrichment of the late photoreceptor regions on the topics 4 and 18 (from the bootstrapped analysis). **g.** Elav staining on the third instar larvae eye-antennal disc (posterior to the morphogenetic furrow, marked with an arrow) of the selected GMR-GAL4 UAS-TF and wild type lines. Scale bar: 20 μm.**h.** Heatmaps showing the normalized coverage of early photoreceptor regions (topic 12) enriched in the GGG motif and late photoreceptor regions (topic 24) enriched in the GGG motif on the ChIP-seq profiles of Prospero, Nerfin-1 and l(3)neo38.

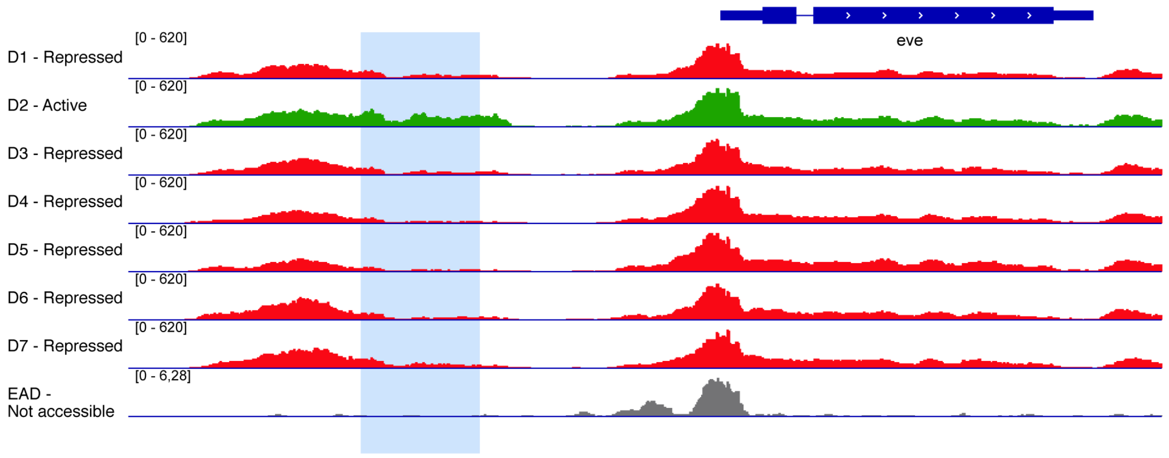

Figure S27. Repressed enhancers show a reduced ATAC-seq signal. Normalized ATAC-seq profiles of the different embryonic domains (D1-7, as described by Bozek *et al.*(Bozek et al., 2019)) and a wild type third instar larvae eye antennal disc profile. The eve stripe 2 enhancer is only active in the second embryonic stripe (D2), while repressed in the rest of the embryo. In the eye-antennal disc this enhancer is neither activated or repressed.

References

Aerts, S., Quan, X.-J., Claeys, A., Naval Sanchez, M., Tate, P., Yan, J., and Hassan, B.A. (2010). Robust target gene discovery through transcriptome perturbations and genome-wide enhancer predictions in Drosophila uncovers a regulatory basis for sensory specification. PLoS Biol. *8*, e1000435.

Aibar, S., González-Blas, C.B., Moerman, T., Huynh-Thu, V.A., Imrichova, H., Hulselmans, G., Rambow, F., Marine, J.-C., Geurts, P., Aerts, J., et al. (2017). SCENIC: single-cell regulatory network inference and clustering. Nat. Methods *14*, 1083.

Ariss, M.M., Islam, A.B.M.M.K., Critcher, M., Zappia, M.P., and Frolov, M.V. (2018). Single cell RNA-sequencing identifies a metabolic aspect of apoptosis in Rbf mutant. Nat. Commun. *9*, 1–13.

Bozek, M., Cortini, R., Storti, A.E., Unnerstall, U., Gaul, U., and Gompel, N. (2019). ATAC-seq reveals regional differences in enhancer accessibility during the establishment of spatial coordinates in the Drosophila blastoderm. Genome Res. *29*, 771–783.

Fritsch, C., Bernardo-Garcia, F.J., Humberg, T.-H., Mishra, A.K., Miellet, S., Almeida, S., Frochaux, M.V., Deplancke, B., Huber, A., and Sprecher, S.G. (2019). Multilevel regulation of the glass locus during Drosophila eye development. PLOS Genet. *15*, e1008269.

Jacobs, J., Atkins, M., Davie, K., Imrichova, H., Romanelli, L., Christiaens, V., Hulselmans, G., Potier, D., Wouters, J., Taskiran, I.I., et al. (2018). The transcription factor Grainyhead primes epithelial enhancers for spatiotemporal activation by displacing nucleosomes. Nat. Genet. *50*, 1011–1020.

Jory, A., Estella, C., Giorgianni, M.W., Slattery, M., Laverty, T.R., Rubin, G.M., and Mann, R.S. (2012). A Survey of 6,300 Genomic Fragments for cis-Regulatory Activity in the Imaginal Discs of Drosophila melanogaster. Cell Rep. *2*, 1014–1024.

McGinnis, C.S., Murrow, L.M., and Gartner, Z.J. (2019). DoubletFinder: Doublet Detection in Single-Cell RNA Sequencing Data Using Artificial Nearest Neighbors. Cell Syst. *8*, 329-337.e4.

Potier, D., Davie, K., Hulselmans, G., Naval Sanchez, M., Haagen, L., Huynh-Thu, V.A., Koldere, D., Celik, A., Geurts, P., Christiaens, V., et al. (2014). Mapping Gene Regulatory Networks in Drosophila Eye Development by Large-Scale Transcriptome Perturbations and Motif Inference. Cell Rep. *9*, 2290–2303.

Stuart, T., Butler, A., Hoffman, P., Hafemeister, C., Papalexi, E., Mauck, W.M., Hao, Y., Stoeckius, M., Smibert, P., and Satija, R. (2019). Comprehensive Integration of Single-Cell Data. Cell *177*, 1888-1902.e21.
